# Isolation of a Cyclic Trinuclear Gold(I) Complex with Metalated Phosphorus Ylides: Synthesis and Structural Properties

**DOI:** 10.1021/acs.inorgchem.3c03740

**Published:** 2024-03-05

**Authors:** Renso Visbal, Noelia Rosado, Jhon Zapata-Rivera, M. Concepción Gimeno

**Affiliations:** † Facultad de Ciencias Naturales y Exactas, Departamento de Química, Universidad del Valle, A.A. 25360 Cali, Colombia; ‡ Centro de Excelencia en Nuevos Materiales (CENM), Universidad del Valle, A.A. 25360 Cali, Colombia; § Departamento de Química Inorgánica, Instituto de Síntesis Química y Catálisis Homogénea (ISQCH) CSIC-Universidad de Zaragoza, 50009 Zaragoza, Spain; ∥ Departamento de Química, Facultad de Ciencias, Universidad de los Andes, Cra 1 #18A-12, A.A. 111711 Bogotá, Colombia

## Abstract

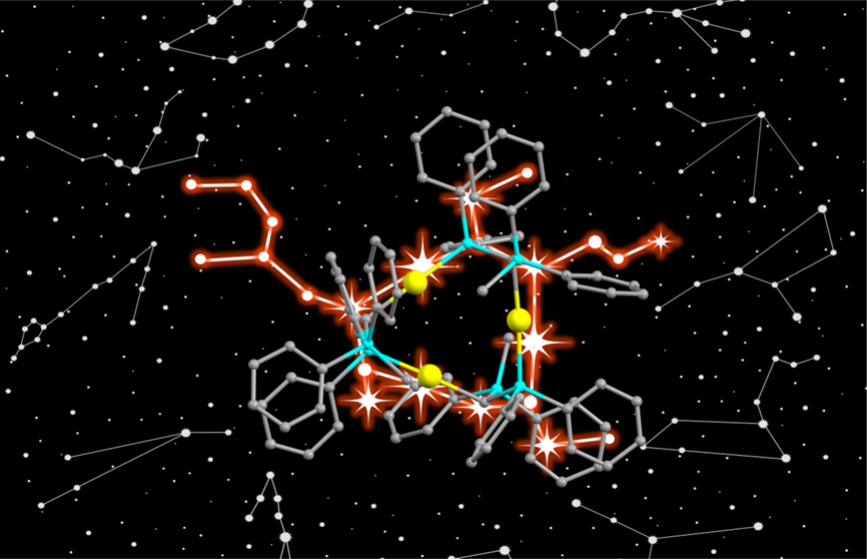

The first chiral and luminescent cyclic trinuclear gold(I) complex, [{AuCH(PPh_2_Me)(Ph_2_P)}_3_]^3+^, has been isolated with metalated phosphorus ylides (PY). This complex was initially obtained through the reaction of either mononuclear [C_6_F_5_SAuCH(PPh_2_Me)(Ph_2_P)]OTf or dinuclear [C_6_F_5_S{AuCH(PPh_2_Me)(Ph_2_P)}_2_](OTf)_2_ thiolate-gold-phosphane complexes in the presence of NaH, followed by the abstraction of the thiopyridine moiety employing either AgOTf or [Cu(CH_3_CN)_4_]PF_6_. Our quest for a more efficient synthesis route led to the development of a streamlined one-pot synthesis method, employing Ag(acac) as both a halogen abstractor and a base, offering a quicker and more direct path to this intriguing trimer. Comprehensive computational studies have unveiled the luminescent characteristics of this complex, which can be attributed to phosphorescence. These emissions originate from ligand-to-metal (LMCT) and metal-centered (MC) charge transfer excited states. Furthermore, the structural analysis via X-ray crystallography corroborated the formation of a trimeric species, featuring three monomers with the [AuCH(PPh_2_Me)(Ph_2_P)] motif. Each monomer exhibits a single chiral center, leading to four possible absolute configurations (RRR, RRS, RSR, and SRR). NMR and X-ray spectroscopy have provided valuable insights, establishing that the former configuration (RRR) is disfavored due to steric hindrance, while the three remaining configurations can interconvert, arising from the structural arrangement of the metallacycle and inherent symmetry operations.

## Abstract 2

The first chiral and luminescent cyclic trinuclear ylide gold(I) complex, [{AuCH(PPh_2_Me)(Ph_2_P)}_3_]^3+^, was synthesized through a one-pot synthesis method utilizing Ag(acac). This innovative approach offers a quicker route to the intriguing trimer, demonstrating its potential as a valuable synthetic route. Comprehensive computational studies revealed the luminescent characteristics, with emissions attributed to ligand-to-metal (LMCT) and metal-centered (MC) charge transfer excited states. X-ray structural analysis confirmed the trimeric species formation, showcasing unique chiral configurations.

## Introduction

**Figure 1:**
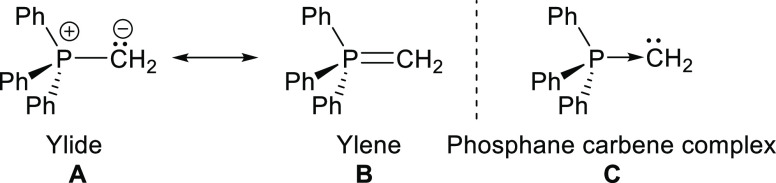
Bond situation in phosphorus ylide ligands.

Phosphorus ylides (PY) are important inorganic and organometallic ligands in the field of coordination chemistry, applied with both transition and nontransition elements. These particular ligands have been described as a carbanion directly bonded to a phosphorus atom with a high degree of formal positive charge, such as the parent methylene-triphenylphosphorane (Ph_3_P=CH_2_ (**A**) Figure 1).^1,2^ It is well-established that the replacement of hydrogen atoms along the backbone with diverse alkyl or aryl groups allows for precise modulation of the electronic and steric properties. Consequently, this fine-tuning enables the controlled reactivity of the respective ylide. Throughout the years, the electronic structure of PYs has been elucidated as residing at the energetic equilibrium between its ylide form (**A**) and its ylene form (**B**), as illustrated in Figure 1.

Recent computational studies have stated that the bonding situation in these ligands can also be interpreted in terms of donor–acceptor interactions between closed-shell ligands and a carbon atom that has two lone-pair orbitals (**C** in Figure 1).^3,4^ In particular, the substitution of hydrogen atoms by another electron-donor atom, such as phosphorus, could provide multiple bonding modes toward acidic metal centers.^5,6^

**Figure 2:**
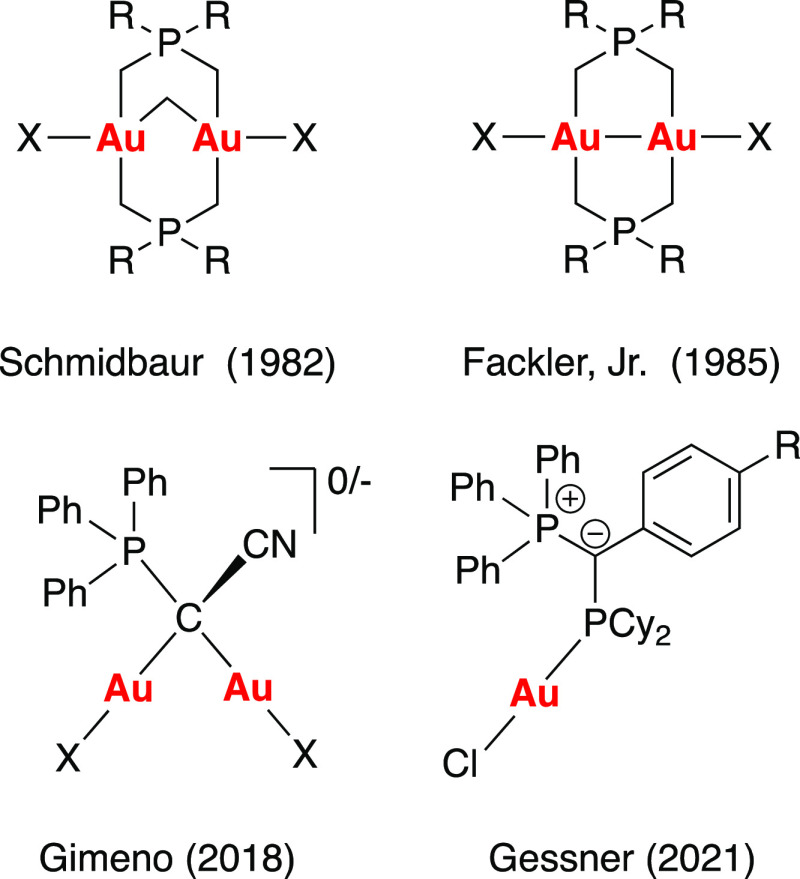
Some phosphorus-ylide- and ylide-substituted phosphane gold complexes.

In this context, the stability of PYs has been used to prepare a great variety of organometallic complexes. Some of the first examples of phosphorus ylide gold complexes published in the literature are those reported by Schmidbaur and Fackler Jr., and co-workers in 1982 and 1985, respectively (Figure 2). In particular, gold complexes have been extensively studied due to their interesting redox potential properties.^7,8^ Recently, the corresponding dinuclear derivatives obtained from the replacement of the halogen atoms by acetonitrile molecules have proven to be very efficient as Lewis catalysts for Mukaiyama addition and alkyne hydroamination reactions.^9^

More recently, capitalizing on the noted stability of PYs, Gimeno and co-workers successfully synthesized a range of coinage metal complexes featuring triphenylphosphonium cyanomethylide or cyanomethyldiide ligands. Notably, certain gold complexes exhibited cytotoxic activity against the human lung carcinoma cell line (A549), surpassing the observed effects of Cisplatin (Figure 2).^10,11^ The electronic and steric properties of PYs can be further fine-tuned by incorporating various alkyl or aryl groups, thereby enhancing their initial characteristics. For instance, Gessner and colleagues synthesized a range of ylide-substituted phosphane gold(I) complexes, which proved to be highly efficient catalysts for the hydroamination of alkynes (Figure 2).^12^

**Figure 3:**
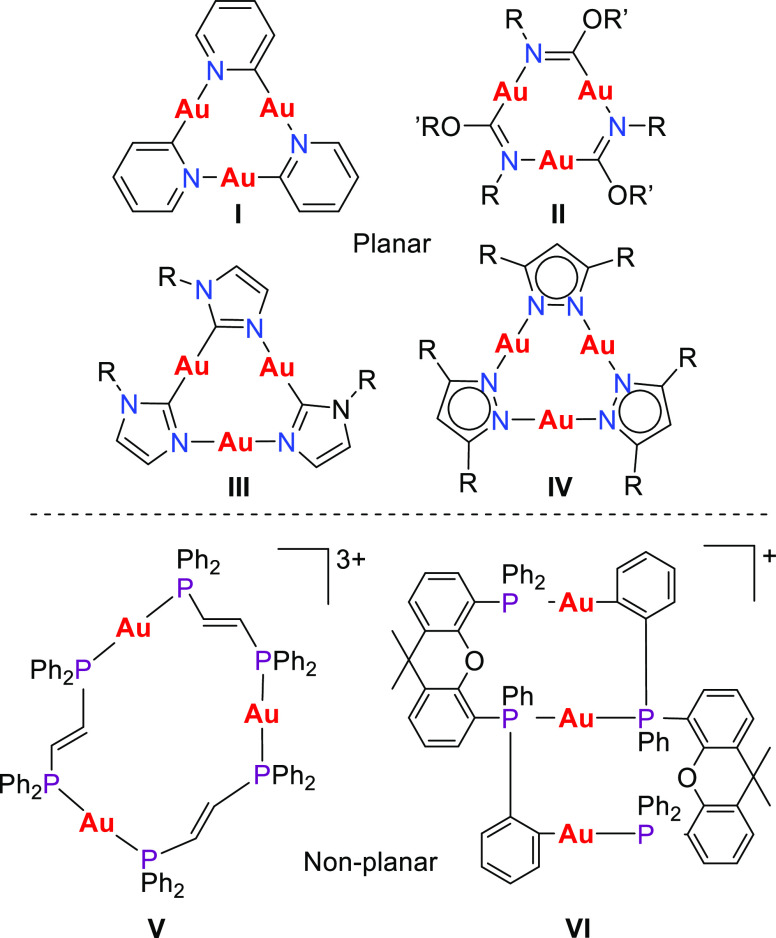
Cyclic trinuclear gold(I) complexes were previously reported.

Cyclic trinuclear complexes (CTCs) of gold(I) containing heterocyclic ligands such as pyridinate (**I**), carbeniate (**II**), imidazolate (**III**), or pyrazolate (**IV**) are an interesting class of metal derivatives which normally display planar metallacycle aggregates (Figure 3). Compound **I**, which is the first example of a CTC gold(I) species reported in the literature,^13^ can form photoluminescent materials through self-associated molecules by aurophilic interactions.^14^ These photoluminescent properties of CTCs can be modulated by external stimuli. For example, the Au···Au contacts present in the aurophilic interaction phenomena observed for complexes **II** can be affected by the presence of electron acceptors, such as solvent molecules, heat, or even mechanical stress (Figure 3).^15−17^

Although Burini and co-workers first reported the formation of a gold(I)-CTC derived from an imidazolate ligand (**III**, R = −Me, −CH_2_Ph in Figure 3),^18^ was not until 2017 when Omary and co-workers reported the shortest pairwise intermolecular aurophilic interactions among all CTCs of gold(I) reported to that date (**III**, R = −Et in Figure 3). Such a complex displayed interesting thermochromic properties as a consequence of internal conversion between energy bands located at 409 and 700 nm below and above 200 K, respectively.^19^ Taking advantage of the high π-basicity of CTCs as a result of the electron-rich π-system,^20^ Esser and co-workers evaluated the properties of CTCs (**I**–**IV** in Figure 3) as donor electron together with bridged naphthalene diimine-based acceptor to form an aromatic donor–acceptor interaction model.^21^ Very recently, Galassi, Manca, and co-workers studied the reactivity of two *N*-alkyl imidazolyl CTCs toward oxidation reactions both experimentally and theoretically. The results point out a new interpretation of the Au(I)–ligand bonding in the oxidized CTCs.^22^

Pyrazolate-based CTCs (**IV** in Figure 3) have also received particular attention. Omary and co-workers investigated the influence of metallophilic interactions by using coinage metals (R = −CF_3_, M = Cu, Ag, Au). These differences were reflected in the phosphorescent properties, accompanied by a change in luminescence thermochromism.^23^ Recently, the same compounds were used to prepare supramolecular assemblies involving the capture of the buckminsterfullerene C_60_ by means of the corresponding dimer of **IV** (R = −CF_3_).^24^

On the other hand, nonplanar CTCs have been less studied than their planar counterparts. In 2012, Bhargava and co-workers reported the synthesis of CTC (**V** in Figure 3) containing the 1,2-bis(diphenylphosphino)ethylene ligand. The optical properties together with computational calculations allowed them to assign the origin of the emissions to metal-perturbed intraligand ^3^IL (phosphane) excited states.^25^ Later, compound **VI** was unexpectedly obtained by thermolysis of the corresponding dinuclear gold precursor (Figure 3).^26^ In this case, X-ray diffraction analysis demonstrated that the gold centers form a linear chain through Au···Au contacts of 2.783(2) and 2.850(1) Å. In addition, complex **VI** displayed strong sky-blue luminescence at 500 nm in both, solid state, and solution, which could be attributed to triplet metal–metal (^3^MM) transition partially mixed with a ligand-to-metal–metal charge transfer (^3^LMMCT) transitions. In this work, the isolation of the first chiral cyclic trinuclear complex containing the [AuCH(PPh_2_Me)(Ph_2_P)] fragment is reported.

## Results and Discussion

### Synthesis of the Cyclic Trinuclear Complex Containing Gold(**I**)-PY Species

**Scheme 1:**
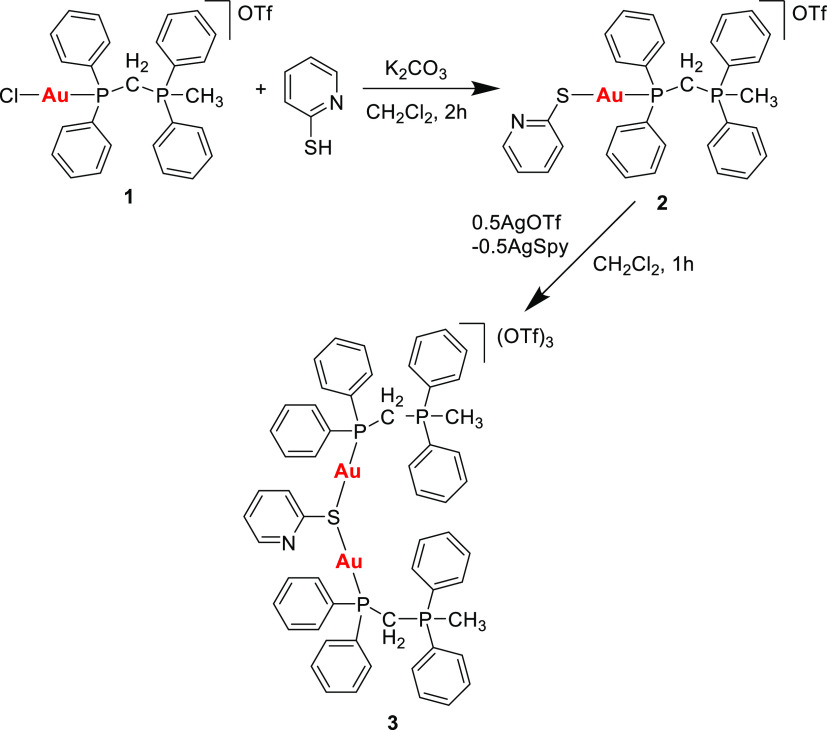
Synthesis of Gold(I) Phosphonium Complexes **2** and **3**

Compound [AuCl(PPh_2_CH_2_PPh_2_Me)]OTf (**1**) was prepared according to the modified procedure reported by some of us.^27^ The reaction of compound **1** with 2-mercaptopyridine in the presence of K_2_CO_3_ afforded the thiolate gold complex **2** as a white solid in a good yield (Scheme 1).

As expected, the main difference of complex **2** compared to complex **1** is the appearance of peaks in the ^1^H NMR spectrum corresponding to the pyridine group (see Figures S1 and S4). Additionally, the protons of the methyl and methylene groups appear as a doublet and a pseudotriplet at 2.66 and 4.62 ppm, respectively, because of the coupling with phosphorus atoms. Consequently, in the ^31^P{^1^H} NMR spectrum two doublets at 21.52 and 22.39 ppm, associated to the phosphonium and the phosphane moieties are observed, respectively (see Figure S5). As observed in Scheme 1, the addition of 0.5 equiv of AgOTf promotes the precipitation of a yellow solid corresponding to [Ag_6_(μ-SPy)_6_]^28^ and the subsequent formation of the dinuclear derivative **3**. Unlike the ^1^H NMR spectrum of complex **3**, in which no significant differences were observed (see Figure S7), the ^31^P{^1^H} NMR spectrum shows a broad signal corresponding to the phosphane ligand directly bonded to the gold center (see Figure S8). This behavior could be associated to some fluxional process on the bonding situation of the sulfur atom (Au–S–Au). In addition to the signal at 78.79 ppm in the ^19^F NMR spectrum corresponding to the trifluoromethanesulfonate anion (see Figure S9), a characteristic peak of complex **3** could be identified in the ESI^+^ mass spectrum with a *m*/*z* = 434.0 assigned to the [μ-pyS(AuPPh_2_CH_2_PPh_2_Me)_2_]^3+^ fragment (see Figure S11).

**Scheme 2:**
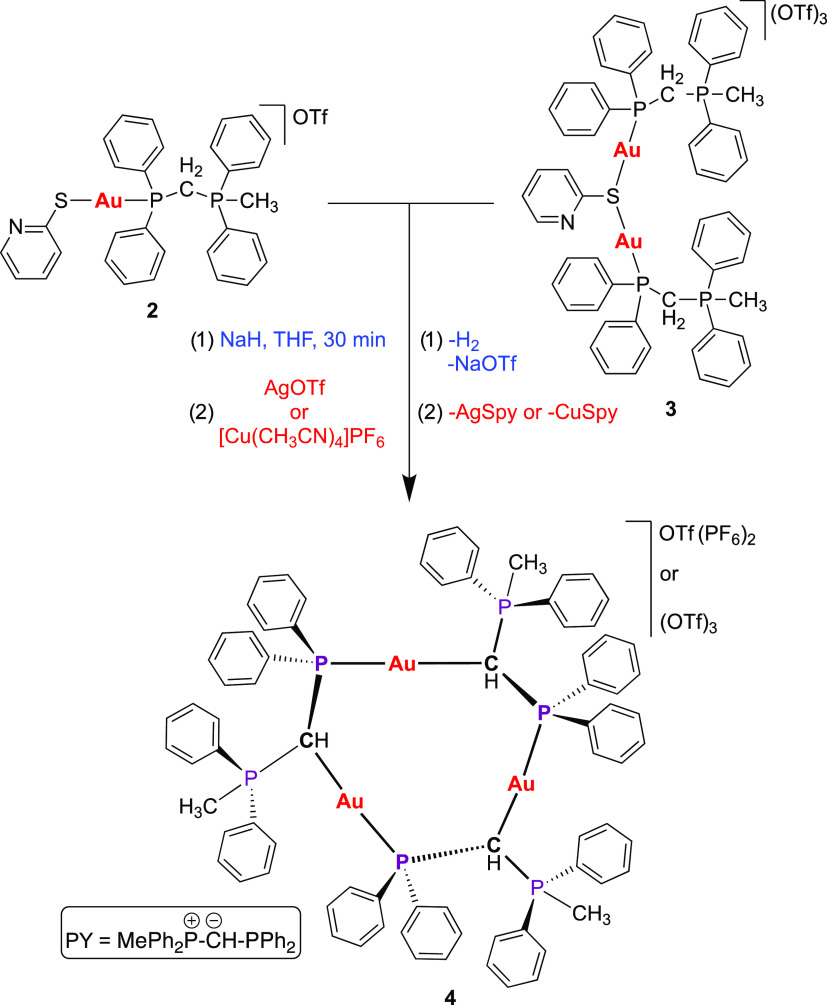
Synthesis of the Trinuclear Gold(I) Metallacycle **4** Containing the Phosphorus Ylide (PY) Moiety

Although both complexes (**2** and **3**) contain a substituted phosphane ligand, these can be conveniently deprotonated in the presence of NaH in dried THF to form the corresponding phosphorus ylide-substituted phosphane gold(I) complexes (see Scheme 2). However, both species decomposed to metallic gold at room temperature probably due to the presence of a strong base such as NaH. Nevertheless, in both cases, the rapid addition of AgOTf or [Cu(CH_3_CN)_4_]PF_6_ promotes the elimination of the S-py moiety, leaving the gold center “naked” and ready to be trapped for another electron-donating ligand.

**Figure 4:**
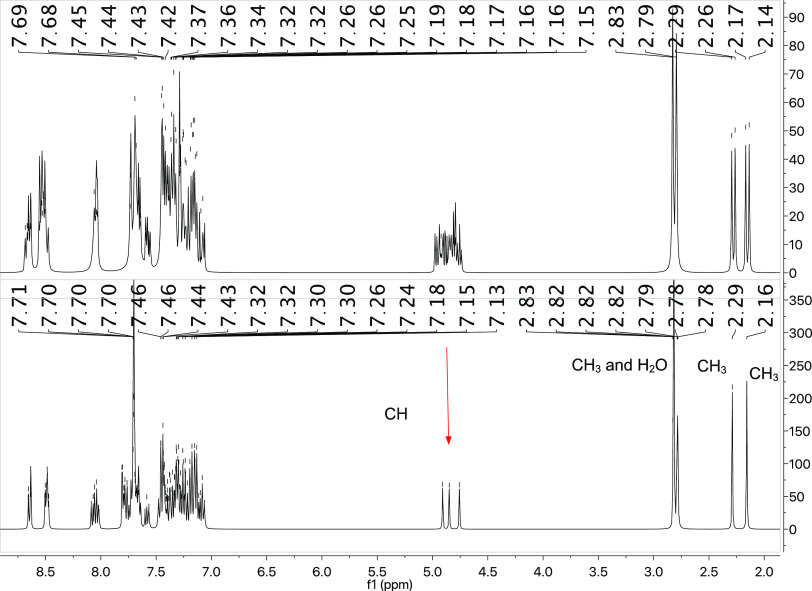
^1^H (top) and ^1^H{^31^P} NMR spectra of complex **4**.

**Figure 5:**
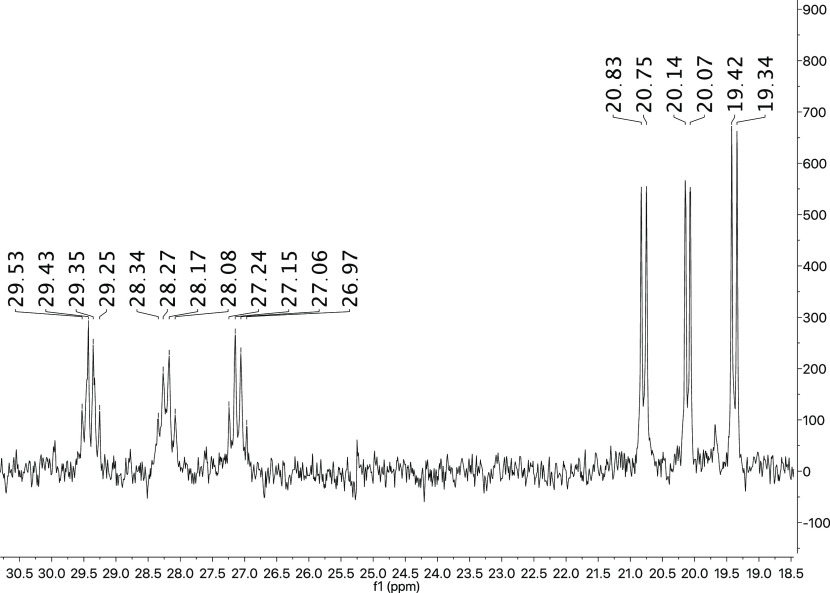
^31^P{^1^H} NMR spectrum of complex **4**.

Surprisingly, the unsaturated gold(I) species is trapped by the PY ligand of another molecule in the reaction mixture, in which the gold center is coordinated by a third PY molecule, thus forming the trinuclear gold(I) complex **4** containing (OTf)_3_^3–^ or OTf(PF_6_)_2_^3–^ anions, depending on the metallic salt, silver or copper, used in its synthesis. Due to the poor solubility of complex **4** containing triflate as counterion, only the (OTf)(PF_6_)_2_ salt could be characterized by NMR spectroscopy. It is noteworthy that each of the PY moieties are nonequivalent, and this can be observed in ^1^H NMR spectrum of derivative **4** (top in Figure 4). As observed, the methyl groups of each phosphonium species appear as separated doublets between 2.81 and 2.16 ppm with *J*_H–P_ ≈ 12 Hz. Interestingly, each proton of the nonequivalent methylene groups is coupled not only with the phosphorus atom of the corresponding phosphonium moiety but also with each phosphorus atom that conforms to the nonplanar trinuclear metallacycle. Thus, these protons appear as multiplets between 4.92 and 4.79 ppm. The nonequivalence of each PY ligand can be rationalized because of the presence of three chiral centers in the CTC. This hypothesis could be corroborated by the ^1^H{^31^P} NMR spectrum (bottom in Figure 4), in which the methyl and methylene groups now appear as singlets. Accordingly, the ^31^P{^1^H} NMR spectrum shows two groups of signals (see Figure 5). The first group, which appears as doublets between 20.79 and 19.38 ppm with *J*_P–P_ ≈ 13.0 Hz, is assigned to the phosphonium moiety, and the second group displays three virtual quartets (vq) between 29.44 and 27.10 ppm with an average *J*_P–P_ ≈ 14.0 Hz is assigned to the phosphorus ylide of the metallacycle.^29^

Matrix-assisted laser desorption ionization–time of flight (MALDI-TOF) mass spectra of both salts of complex **4** were identified. For example, complex **4** containing only the CF_3_SO_3_^–^ anion was identified with the peak with *m*/*z* = 2083.5 and an isotope pattern corresponding to the fragment [AuCH(PPh_2_Me)(Ph_2_P)]_3_(OTf)_2_^+^ or [M-OTf]^+^. Similarly, complex **4** bearing [(PF_6_)_2_OTf]^−3^ anions was also detected in the MALDI^+^-TOF spectrum with a *m*/*z* = 2079.3 assigned to the [M-PF_6_]^+^ peak (see Figures S16 and S17, and Experimental Section).

**Figure 6:**
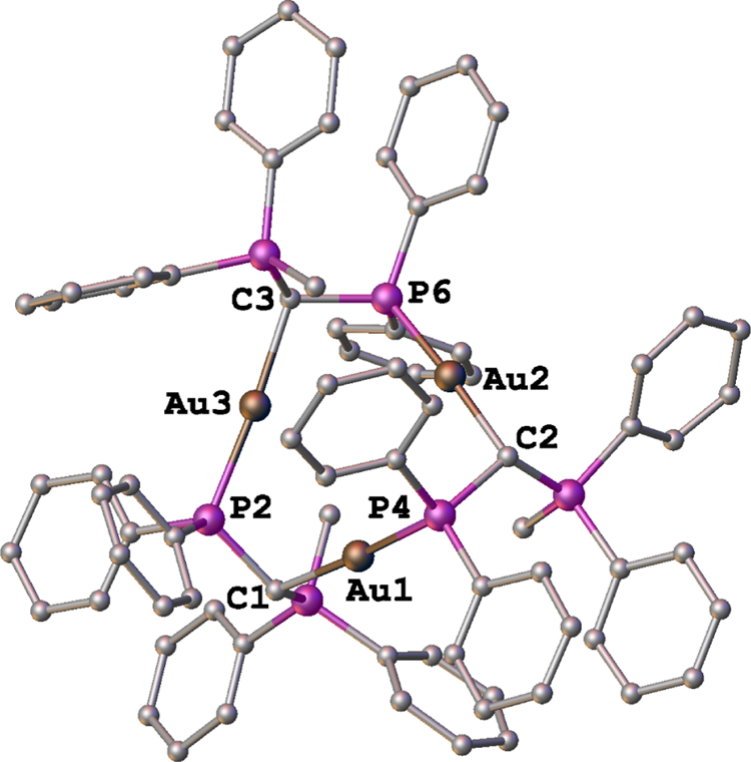
Molecular structure of the trinuclear [AuCH(PPh_2_Me)(Ph_2_P)]_3_^3+^ cation of complex **4**. Hydrogen atoms, anions, and solvent molecules are omitted for clarity.

Suitable crystals of CTC **4** containing [(PF_6_)_2_OTf]^−3^ were obtained and characterized by X-ray diffraction analysis, and the solid structure is depicted in Figure 6. Complex **4** crystallizes in the space group P-1 with one independent molecule in the asymmetric unit (see Table S1). As expected for gold(I) complexes, the geometry around each of the three metal centers is linear, with C–Au–P bond angles ranging from 173.1(2) to 176.0(2) (see Tables 1 and S3).

**Table 1:** Selected Bond Lengths (Å) and Angles (deg), for complex **4**

compound	4
Au1–C1	2.107(9)
Au2–C2	2.106(8)
Au3–C3	2.101(8)
Au1–P4	2.277(2)
Au2–P6	2.277(2)
Au3–P2	2.284(2)
C1–Au1–P4	175.2(2)
C2–Au2–P6	173.1(2)
C3–Au3–P2	176.0(2)

As expected, Au–C and Au–P bond distances are similar to those found in Au(I)-PY and Au(I)-phosphane complexes previously reported.^12,30^ Unlike planar CTCs of gold(I), complex **4** does not present aurophillic interactions since the interatomic distances calculated were 3.775, 3.759, and 3.572 Å for Au2–Au3, Au1–Au2, and Au1–Au3, respectively. Although the latter is shorter than the others, it is still too long to be considered as a formal Au···Au contact.^31^

**Figure 7:**
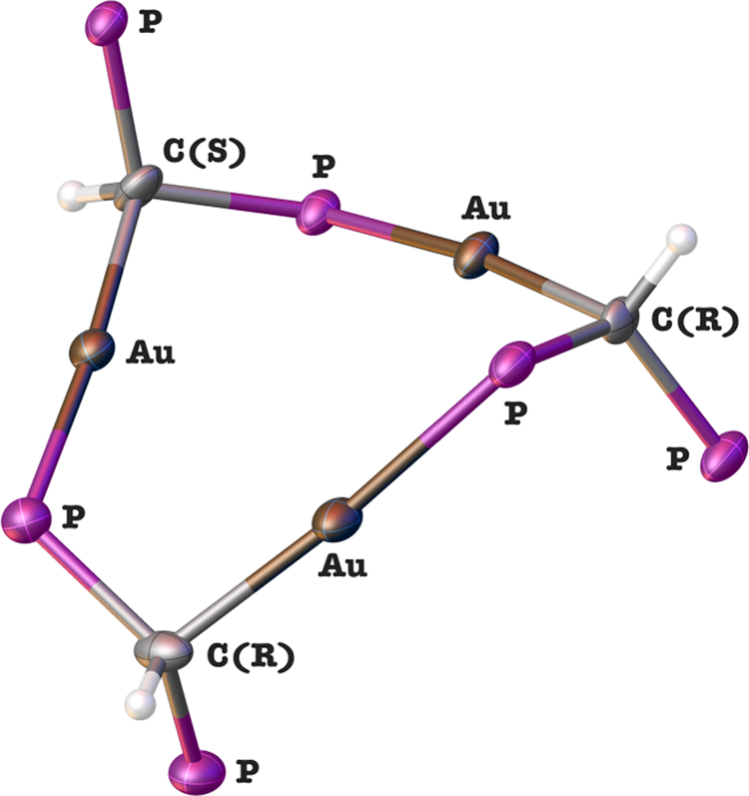
View of trinuclear metallacycle **4** with 50% probability ellipsoids.

A deeper analysis of the crystal structure of complex **4** allows a better understanding of the results obtained from the NMR spectroscopy previously commented. In order to get a better view of the tricationic trinuclear metallacycle, phenyl, and methyl groups from CTC **4** have been omitted. The resulting structure is depicted in Figure 7. A visual inspection shows that the 9-membered metallacycle contains three chiral centers which confer an RRS absolute configuration. Additionally, since complex **4** crystallized in the *P*1̅ space group and a racemic mixture was obtained, stereoisomer SSR must be present in the crude reaction. It is noteworthy, despite all of the carbon atoms having the same connectivity, due to the presence of three chiral centers together with the nonplanarity of the metallacycle, each of the [AuCH(PPh_2_Me)(Ph_2_P)] fragments is inequivalent with respect to each other in the metallacycle. This fact is consistent with the presence of three signals for the PY-substituted ligand in both the ^1^H and ^31^P{^1^H} NMR spectra previously commented. In this context, there are four possible diastereoisomers, namely, RRS, RSR, SRR, and RRR. The latter is sterically unfavored and therefore not observed in the ^1^H nor ^13^C NMR spectra. By symmetry operations, each of these can lead to SSR, SRS, and RSS configurations, that is, the corresponding enantiomer, for a total of six absolute configurations, with all of them indistinguishable by NMR spectroscopy. On the other hand, if the RRR isomer and its corresponding enantiomer (SSS) were present, they would be distinguishable from RRS by NMR because of the existence of a C3 rotation axis in the former.

**Scheme 3:**
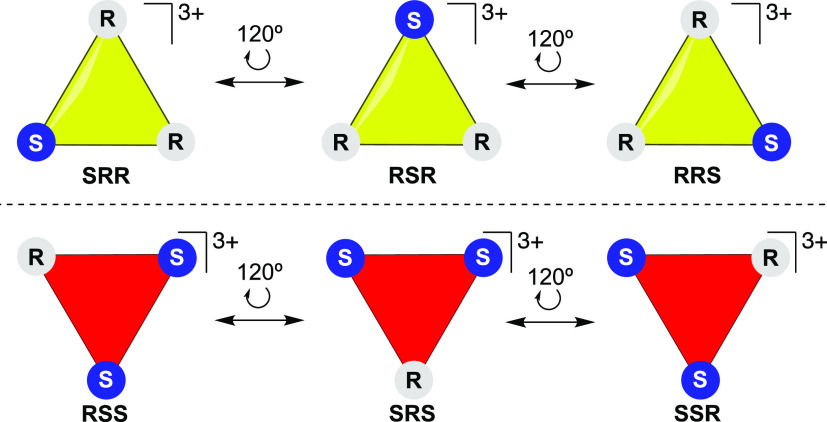
Six of the Eight Possible Configurations and Their Interconversion in Each Enantiomer Group

However, it is noteworthy that due to the cyclic nature of the trinuclear metallacycle, each of these configurations can interconvert. That is, SRR ↔ RSR ↔ RRS and SSR ↔ SRS ↔ RSS are rotated 120° about an axis perpendicular to the plane arising from the three chiral centers, as each monomer has the same substituent groups. This leads to a reduction of the three possible configurations observable experimentally for each enantiomer group to only one (Scheme 3). Crystal packing of the molecule points out the presence of both inter- and intramolecular interactions. In fact, some of the phenyl groups of different phosphane ligands of complex **4** present π···π stacking interactions with interplanar angles in the range from 5.6 to 10.63°, and centroid-centroid distances of around 3.595 Å.^32^

**Scheme 4:**
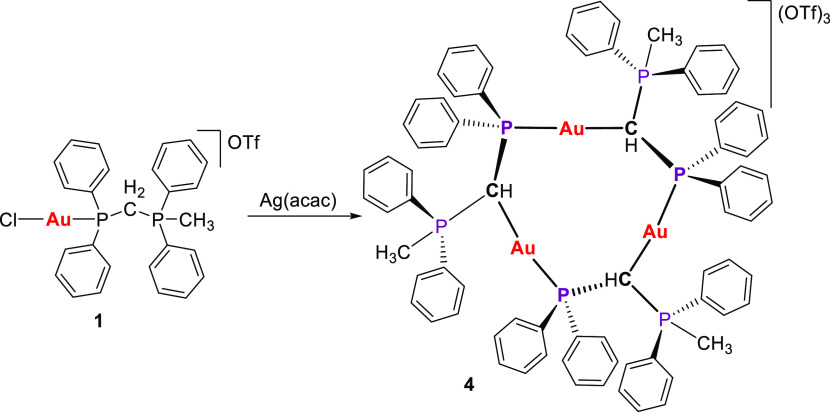
Straightforward Synthesis of the Trinuclear Gold(I) Metallacycle **4**

Considering the intriguing structural framework, we sought a more straightforward pathway for its formation. Consequently, after attempting the reaction of the complex [AuCl(PPh_2_CH_2_PPh_2_Me)]OTf (**1**) with several bases, we obtained the desired complex in an impure form. Interestingly, the reaction of complex **1** with Ag(acac) results in both deprotonation of the phosphane-phosphonium moiety and abstraction of the chloride ligand, thereby affording compound **4** through a faster pathway (Scheme 4).

### Photophysical Properties and Computational Studies

**Figure 8:**
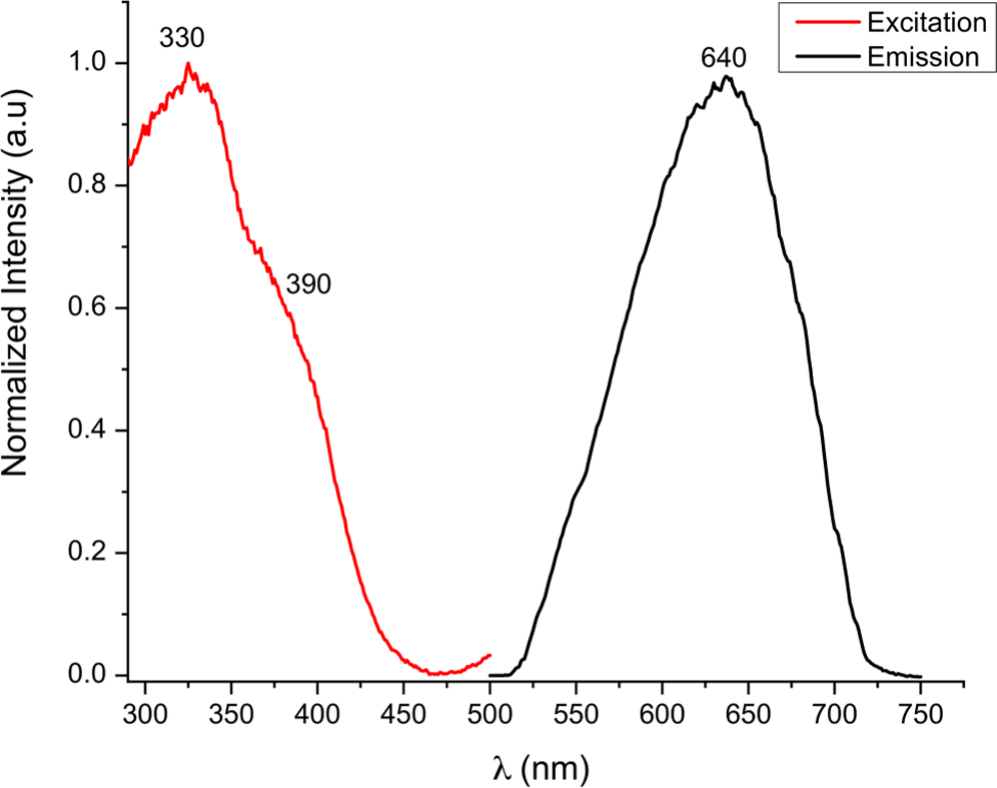
Excitation (left) and emission (right) spectra of complex **4** in acetone (0.5 mg/25 mL).

The UV–vis spectra of complexes **2** and **3** have been measured for comparison purposes (Figures S19 and S20) and show absorption maxima at 225 and 280 nm and 225 and 305 nm, respectively. According to similar compounds previously reported in the literature, these bands can be attributed to π → π* or *n* → π* transitions with a major contribution from the phenyl and the pyridine rings.^27,30^ A visual inspection of the electronic spectrum of complex **4** allows us to corroborate the presence of two bands at around 230 and 350 nm (Figure S21). As observed, the latter is red-shifted, with respect to those obtained for complexes **2** and **3**. This may be indicative that the absorption band at around 225 nm observed for all of the complexes can be tentatively assigned to originate from intraligand (IL) transitions. On the other hand, the second one between 300 to 350 nm is expected to have an important contribution from the metal center, which could be associated with a ligand-to-metal (LMCT) charge transfer excited states. Despite mono- and dinuclear complexes being nonemissive species, CTC **4** is a strong orange-red emitter showing an emission maximum at around 640 nm after irradiation at 330 or 390 nm in acetone at room temperature (Figure 8).

The emission lifetime measured for complex **4** in acetone was found to be 23.89 μs (see Figure S22 from Supporting Information), together with the orange–red emission observed suggests a phosphorescent nature. This could be in good agreement with an important contribution of the metal d orbitals to the electronic transitions involved in the excited states.

**Figure 9:**
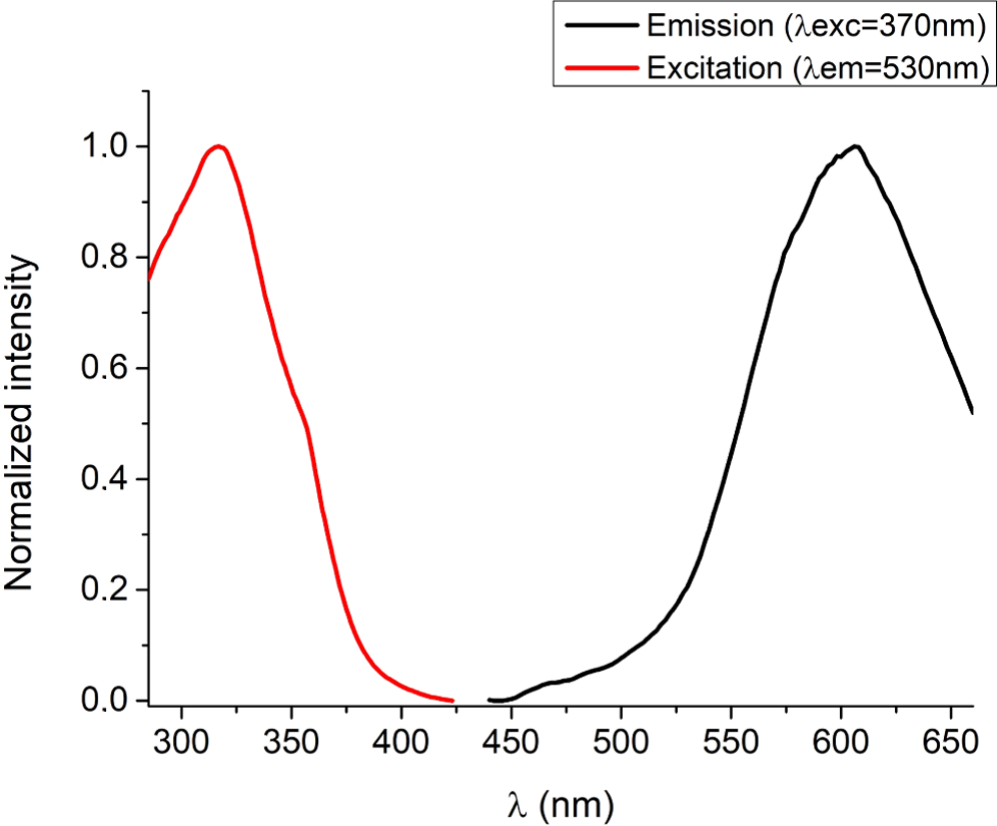
Excitation (left) and emission (right) spectra of complex **4** in the solid state at 77 K.

Complex **4** is also emissive in the solid state, both at room temperature and 77 K. The complex exhibits a broad emission band with a maximum of around 600 nm, both at room temperature and at 77 K (Figures 9 and S23), which could be attributed to a mixture of LMCT and MC transitions, as has been corroborated by theoretical calculations (see below). Lifetimes in the solid state at room temperature are 5.22 and 16.66 μs and, 17.64 and 41.22 μs at 77 K (S24–25), indicating the phosphorescence nature of the emissions.

**Figure 10:**
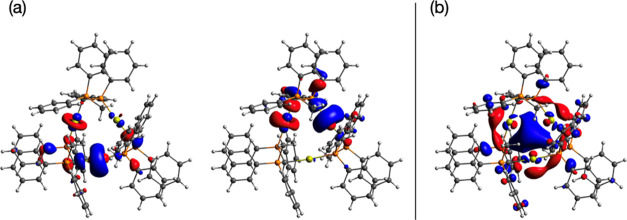
Frontier molecular orbitals of CTC **4** (a) nearly degenerate HOMO–1 and HOMO and (b) LUMO.

To in-depth understand the optical properties of CTC **4**, several computational studies were performed on the framework of density functional theory (DFT).^33^ In all calculations the PBE0 functional was used (computational details are described in the Supporting Information); this functional has shown trustable results in the description of comparable gold(I) complexes.^34^ First, we studied the electronic structure of the singlet ground state by means of a single-point calculation on the geometry solved by X-ray diffraction (XRD). The analysis of the frontier molecular orbitals shows that the highest occupied molecular orbital (HOMO) and HOMO–1 are centered on the bridging Au atoms, particularly on the 5d_*z*^2^_ orbitals (Figure 10). The lowest-unoccupied molecular orbital (LUMO) is seen to be delocalized around the central ring of the molecule with a larger contribution of the gold 6p orbitals. Moreover, the higher energy unoccupied orbitals are largely delocalized on the phenyl moieties of the PPh_2_CH_2_PPh_2_Me ligands (Figure S27). Second, using the TD-DFT approach, the absorption wavelengths have been estimated by means of the energy calculation of the low-lying excited singlets. At the outset, geometry optimization calculations at the singlet ground state were performed on **4**, with no significant changes regarding the geometry and electronic structure described above. Then, the singlet excited-state energies and oscillator strengths were calculated at the same level of theory. We have depicted the calculated absorption spectrum (Figure S26), which is in good agreement with the UV–vis absorption bands (a shifting lower than 50 nm). As outlined in Table 2, the broadband around 350 nm in the UV–vis spectrum is mainly associated with the population of the sixth (325.1 nm) and 14th (315.5 nm) excited singlets. The analysis of the electronic structure of these excited states reveals that the absorption wavelength at 325.1 nm is essentially the result of an electron transition from a combination of Au 5d_*z*^2^_ orbitals (5d_*z*^2^–H_ or HOMO) to the bonding combination of Au 6p orbitals (6p_σ_ or LUMO, see Figure S28). The wavelength at 315.5 is mainly a transition from phenyl moieties to the Au 6p_σ_ orbital, and the nature of these transitions is found to be mainly metal-centered (MC) and ligand-to-metal charge transfer transitions (LMCT), respectively. Traditionally, the former assignment (MC) is associated with the presence of aurophilic interactions,^35,36^ and this could be in disagreement with the absence of formal Au···Au contacts in complex **4**. However, the gold–gold distances found (3.775, 3.759, and 3.572 Å) are not significantly different from the expected range of distances for this phenomenon (2.8–3.5 Å).^31^ Additionally, the phosphorescent nature of the emissions, together with the strong electronic correlation in the metallacycle observed in the ^31^P NMR spectrum of **4** points to a significant contribution from the gold center and, therefore, to MC transitions. The band at 265 nm in the UV–vis region is mainly consistent with calculated absorption wavelengths at 292.7 and 290.4 nm, which have been characterized mostly as π_Ph_ → π_Ph_ and 5d_*z*^2^_ → π_Ph_. Thus, these wavelengths arise from transitions of ligand-to-ligand (LL) and metal-to-ligand (ML) nature (Table 2). Regarding the band at 230 nm (largely associated with the excited singlets at 279.5 and 279.1 nm in the calculated absorption spectrum), it is mainly an ensemble of LMCT transitions, namely, transitions from the phenyl moiety to the gold ring, labeled as π_Ph_ → 6p_σ_ in Table 2. Finally, at the optimized geometry for the triplet state, we have carried out a rough estimation of the phosphorescence wavelength by the evaluation of the triplet-singlet (S-T) energy difference. We have found a S-T gap of 66.7 kcal mol^–1^, which is in line with an emission wavelength of 428.6 nm. The visible deviation with regard to the observed red emission lies in the fact that a more accurate computational description requires to consider vibronic and spin–orbit couplings, which currently due to the size of the system is beyond our computational capabilities. The electronic structure analysis of the singlet and triplet states reveals that the emission wavelength is the result of an LMCT from a phenyl π_Ph_ orbital to the Au 5d_*z*^2^_ orbitals (Figure S28). In fact, the energy difference between these orbitals (68.6 kcal mol^–1^) is comparable to the S-T gap, demonstrating the pivotal role of gold atom 5d orbitals in both absorption and emission phenomena.

**Table 2:** Calculated Wavelengths (nm), Oscillator Strength, Dominant Transitions (%), and Nature of the Excited Singlet States Associated to the Absorption Spectrum of Complex **4**

state^a^	λ_exp_	λ_cal_	oscillator strength	dominant transitions^b^	nature
6^1^A	350	325.1	0.1940	78% 5d_*z*^2^-H_ → 6p_σ_	MC
14^1^A		315.5	0.2104	60% π_Ph-24_ → 6p_σ_	LMCT
72^1^A	265	292.7	0.2643	39% π_Ph-22_ → π_Ph-34_	LLCT
				15% π_Ph-24_ → π_Ph-36_	LLCT
82^1^A		290.4	0.2064	38% 5d_*z*^2^_ → π_Ph-39_	MLCT LMCT
				18% π_Ph-14_ → 6p_σ_	
127^1^A	230	279.5	0.2018	12% π_Ph-11_ → 6p_σ_	LMCT MLCT
				11% 5d_*z*^2^-H1_ → π_Ph-40_	
129^1^A		279.1	0.1500	56% π_Ph-11_ → 6p_σ_	LMCT

a Only included excited singlets with oscillator strengths larger than 0.15.

b Dominant transitions with weight larger than 10%. Orbitals are shown in Figure S27.

## Experimental Section

### General Measurements and Analysis Instrumentation

C, H, and N analyses were carried out with a PerkinElmer 2400 microanalyzer. Mass spectra were recorded on a Bruker Esquire 3000 Plus, with an electrospray (ESI) technique. UV–vis spectra were recorded with 1 cm quartz cells on an Evolution 600 spectrophotometer. Steady-state photoluminescence spectra in solution were recorded with a Jobin-Yvon-Horiba fluorolog FL-3–11 spectrometer using band pathways of 3 nm for both excitation and emission. Phosphorescence lifetimes were recorded using a Fluoromax phosphorimeter accessory containing a UV xenon flash tube at a flash rate of between 0.05 and 25 Hz. The lifetime data were fit using the Jobin-Yvon software package and the Origin 5.0 program. Steady-state and lifetime measurements in the solid state were carried out in a FluoTime 300 (PicoQuant) fluorescence spectrometer. Steady-state spectra were recorded by using a xenon lamp and a 280 nm LED for lifetime studies.

^1^H, ^13^C{^1^H}, ^31^P{^1^H} and ^19^F NMR, including two-dimensional (2D) experiments, were recorded at room temperature on a Bruker Avance 400 spectrometer (^1^H, 400 MHz; ^13^C, 100.6 MHz; ^31^P, 162 MHz; ^19^F, 376.5 MHz) or on a Bruker Avance II 300 (^1^H, 300 MHz; ^13^C, 75.5 MHz; ^31^P, 121.5 MHz; ^19^F, 282.3 MHz) with chemical shifts (δ, ppm) reported relative to the solvent peaks of the deuterated solvent.^37^

### Crystallographic Data

#### Crystal Structure Determinations

Crystals were mounted in inert oil on glass fibers and transferred to the cold gas stream of an Xcalibur Oxford Diffraction diffractometer equipped with low-temperature attachments. Data were collected using monochromated Mo Kα radiation (λ = 0.71073 Å). The scan type was ω. Absorption corrections based on multiple scans were applied using spherical harmonics implemented in SCALE3 ABSPACK scaling algorithm.^35^ The structures were solved with the ShelXS structure solution program using direct methods and by using Olex2 as the graphical interface.^36^

### Materials and Procedures

Complex **1** was prepared according to the published procedure.^27^ All other starting materials and solvents were purchased from commercial suppliers and used as received unless otherwise stated.

#### Synthesis of Complex **1**

0.70 g (1.276 mmol) of the phosphonium salt [PPh_2_CH_2_PPh_2_Me]OTf was added to a solution of 0.4092 g (1.276 mmol) of [AuCl(tht)] in 20 mL of CH_2_Cl_2_. After 2 h of stirring, a green–yellow precipitate was filtered off and the resulting colorless solution was evaporated under vacuum until minimum volume. Following the treatment with diethyl ether, complex **1** was obtained as a white solid. (yield: 80.5%). ^1^H NMR (300 MHz, CDCl_3_, 294 K) δ 7.93–7.74 (m, 10H, Ph, PPh_2_), 7.63–7.56 (m, 4H, Ph, PPh_2_), 7.50– 7.42 (m, 6H, Ph, PPh_2_), 4.62 (dd, *J*_H–P_ = 11.4 Hz, *J*_H–P_ = 15.2 Hz, 2H, CH_2_), 2.45 (d, *J*_H–P_ = 13.5 Hz, 3H, CH_3_). ^31^P–{^1^H} NMR (121.5 MHz, CDCl_3_) δ 21.6 (d, *J*_P–P_ = 4.5 Hz, CH_2_PPh_2_CH_3_), 17.74 (br, AuPPh_2_CH_2_). RMN ^19^F (376.5 MHz, CDCl_3_) δ −78.2.

#### Synthesis of Complex **2**

A flask was charged with 2-mercaptopyridine (0.64 mmol), dichloromethane (15 mL) and an excess of K_2_CO_3_. The mixture was kept under stirring for 1 h, in which a slight diminishing of the yellow color was observed. After filtration over Celite, 0.6 mmol [AuCl(PPh_2_CH_2_PPh_2_Me)]OTf (**1**) was added with stirring for 1 h. During this time, the color of the crude reaction mixture changed to a yellow–green solution. The solvent was removed in vacuo until ca. 2 mL and then the product was precipitated and washed with diethyl ether (2 × 5 mL) as a yellow solid (yield: 92.3%). ^1^H NMR (400 MHz, CDCl_3_, 294 K) δ 8.15 (d, *J*_H–H_ = 4.7 Hz, 1H, H6, py), 8.04–7.98 (m, 4H, Ph, PPh_2_), 7.90–7.84 (m, 4H, Ph, PPh_2_), 7.66–7.62 (m, 2H, H3–H4, py), 7.50–7.33 (m, 12H, Ph, PPh_2_), 6.86 (t, *J*_H–H_ = 6.0 Hz, 1H, H5, py), 4.69 (t, *J*_H–P_ = 12.9 Hz, 2H, CH_2_), 2.72 (d, *J*_H–P_ = 13.4, 3H, CH_3_). ^31^P–{^1^H} NMR (162 MHz, CDCl_3_) δ 22.39 (d, *J*_P–P_ = 9.8 Hz, AuPPh_2_CH_2_), 21.52 (d, *J*_P–P_ = 9.8 Hz, CH_2_PPh_2_CH_3_). ^19^F NMR (376.5 MHz, CDCl_3_) δ −78.19. Elemental analysis for C_32_H_29_AuF_3_NO_3_P_2_S_2_ calcd (%): C 44.92; H 3.42; N 1.64; found (%): C 45.22; H 3.59; N 1.81.

#### Synthesis of Complex **3**

To a solution of the corresponding thiolate gold(I) complex (**2**) (0.117 mmol) in dichloromethane (20 mL) was added AgOTf (0.058 mmol). The mixture was stirred for 1 h protected from light, then filtered through Celite, and the solvent was removed in vacuo to ca. 2 mL. The product was precipitated with diethyl ether (20 mL) and washed again with Et_2_O (2 × 5 mL) to give a white solid (yield: 88.2%). ^1^H NMR (300 MHz, CDCl_3_, 294 K) δ 8.24 (d, *J*_H–H_ = 4.7 Hz, 1H, H6, py), 7.93–7.44 (m, 40H, Ph, PPh_2_ and 2H, H3–H4, py), 7.14–7.10 (m, 1H, H5, py), 4.70 (t, *J*_H–P_ = 12.1 Hz, *J*_H–P_ = 15.1, 4H, CH_2_), 2.51 (d, *J*_H–P_ = 13.2 Hz, 6H, CH_3_). ^13^C–{^1^H} NMR (101 MHz, CD_2_Cl_2_, 294 K) δ 166.11 (s, C6, py), 144.45 (s, C5, py) 135.82 (d, *J*_C–P_ = 3.1 Hz, C*p*, Ph, PPh_2_CH_3_), 134.30 (d, *J*_C–P_ = 15.5 Hz, C*o*, Ph, PPh_2_CH_3_), 133.43 (d, *J*_C–P_ = 2.7 Hz, C*p*, Ph, PPh_2_), 133.21 (d, *J*_C–P_ = 10.6 Hz, C*m*, Ph, PPh_2_CH_3_), 130.71 (d, *J*_C–P_ = 13.1 Hz, C*o*, Ph, PPh_2_), 130.12 (d, *J*_C–P_ = 12.6 Hz, C_*m*_, Ph, PPh_2_), 127.82 (dd, *J*_C–PCH_3__ = 59.8 Hz and *J*_C–P_ = 5.0 Hz, C_ip_, Ph, PPh_2_CH_3_), 119.54 (s, C3–C4, py), 118.29 (dd, *J*_C–P_ = 88.1 Hz and *J*_C–PCH_3__ = 2.4 Hz, C_*ip*_, Ph, PPh_2_), 22.12 (dd, *J*_C–PCH_3__ = 50.2 Hz and *J*_C–P_ = 25.3 Hz, CH_2_), 9.99 (d, *J*_C–P_ = 56.6 Hz, CH_3_), ^31^P–{^1^H} NMR (162 MHz, CD_2_Cl_2_, 294 K) δ 22.02 (s, br), 21.01 (d, *J*_P–P_ = 4.8 Hz). ^19^F NMR (376.5 MHz, CD_2_Cl_2_) δ −78.79. ESI^+^ mass (L = PPh_2_CHPPh_2_Me): *m*/*z* = 434.0 [M-3OTf]^3+^, *m*/*z* = 706.0 [M-AuL-3OTf]^+^, *m*/*z* = 745.0 [M-PySAuL-2OTf]^+^, and *m*/*z* = 856.0 [M-AuL-2OTf + H^+^]^+^ [py-S-AuPPh_2_CH_2_PPh_2_Me]OTf. Elemental analysis for C_60_H_54_Au_2_F_9_NO_9_P_4_S_4_ calcd (%): C 41.18; H 3.11; N 0.80; found (%): C 41.43; H 3.39; N 1.05.

#### Synthesis of Complex **4**

Method 1: A Schlenk was charged with compound **2** or compound **3** (0.35 mmol), an excess of NaH (1 mmol), and dried THF (20 mL). After 45 min of stirring at room temperature, the crude reaction was filtered over Celite, and a colorless solution was obtained. Then, [Cu(CNCH_3_)_4_]PF_6_ or AgOTf (0.35 mmol) was added to the clear solution, and after 30 min under stirring, a yellow precipitate was filtered off over Celite. The filtrate was evaporated until dryness and then redissolved in dichloromethane (2 mL). The addition of diethyl ether (15 mL) afforded complex **4** of the form (OTf)(PF_6_)_2_ (88.8%) or [(μ-Au)PPh_2_CHPPh_2_Me]_3_(OTf)_3_ (93.3%) as white solids, respectively. ^1^H NMR (400 MHz, (CD_3_)_2_CO, 294 K) δ 7.82–7.57 (m, 20H, Ph), 7.47–7.7.06 (m, 40H), 4.96–4.72 (m, 3H, CH), 2.29 (d, *J*_H–P_ = 12.3 Hz, 3H, CH_3_), 2.16 (d, *J*_H–P_ = 12.7 Hz, 3H, CH_3_), 2.29 (d, *J*_H–P_ = 12.3 Hz, 3H, CH_3_). ^1^H–{^31^P} (400 MHz, (CD_3_)_2_CO, 294 K) δ 4.80 (s, 1H, CH), 4.75 (s, 1H, CH), 4.67 (s, 1H, CH), 2.15 (s, 3H, CH_3_), 2.02 (s, 3H, CH_3_), 1.96 (s, 3H, CH_3_). ^31^P NMR (162 MHz, CD_3_)_2_CO, 294 K δ 29.14 (vq, *J*_P–PCH_3__ ≈ *J*_P–PP_ = 13.9 Hz), 28.14 (vq, *J*_P–PCH_3__ ≈ *J*_P–PP_ = 14.7 Hz), 27.07 (vq, *J*_P–PCH_3__ ≈ *J*_P–PP_ = 14.6 Hz), 20.79 (d, *J*_CH_3_P–P_ = 13.0 Hz), 20.11 (d, *J*_CH_3_P–P_ = 12.4 Hz), 19.38 (d, *J*_CH_3_P–P_ = 13.3 Hz). ^19^F NMR (376.5 MHz, (CD_3_)_2_CO, 294 K δ −73.50 (d, *J*_F–P_ = 708.2 Hz, PF_6_^–^), −79.85 (OTf^–^). MALDI^+^ mass (L = PPh_2_CHPPh_2_Me): *m*/*z* = 2079.3 [M-PF_6_]^+^, *m*/*z* = 1933.3 [M-2PF_6_–H^+^]^+^, *m*/*z* = 1189.3 [M-AuL-2PF_6_–OTf-H^+^]^+^, *m*/*z* = 993.3 [AuL_2_^+^], *m*/*z* = 595.1 [AuL]^+^, *m*/*z* = 399.1 [L + H^+^]^+^. Elemental analysis for C_79_H_72_Au_3_F_15_O_3_P_8_S calcd (%): C 42.64; H 3.26; found (%): C 42.90; H 3.45.

#### Synthesis of Complex (OTf)_3_ (**4**)

Method 2: To a solution of compound [PPh_2_CH_2_PPh_2_Me]OTf (55.0 mg, 0.1 mmol) in dry dichloromethane (10 mL) [AuCl(tht)] (32.3 mg, 0.1 mmol) was added under argon atmosphere, After 30 min of reaction, silver acetylacetonate (20.7 mg, 0.1 mmol) was added at the reaction was reacted during 4 h protected from light dark. Then, the solution was filtered by diatomaceous earth, and evaporation of the solvent and addition of diethyl ether afford compound **4** as a white solid (35% yield).

Complex **4** with OTf^–^ as counterion has low solubility and only could be characterized by ^1^H and ^31^P{^1^H} NMR, which is coincident with the (OTf)(PF_6_)_2_ salt and by mass spectrometry MALDI^+^ mass: *m*/*z* = 2083.5 [M-OTf]^+^.

## Conclusions

In conclusion, our research has achieved a significant milestone by successfully isolating the first chiral and luminescent cyclic tricationic trinuclear gold(I) complex, [{AuCH(PPh_2_Me)(Ph_2_P)}_3_]^3+^, containing a metalated phosphorus ylides (PY) moiety. This complex was synthesized through a multistep process, involving the initial reaction of either mononuclear or dinuclear thiolate-gold-phosphane complexes, followed by the selective removal of the thiopyridine moiety. To streamline and expedite the synthesis, we devised a one-pot methodology that involves the use of Ag(acac) as both a halogen abstractor and a base, resulting in a more efficient and direct route to this intriguing tricationic trimeric complex. The results obtained from the characterization by means of spectroscopic and spectrometric techniques such as NMR, MS spectrometry, elemental analysis, and UV–vis suggest the formation of a trimeric species that contains three chiral centers. The structural arrangement in both solution and solid state of the metallacycle supports the idea of the existence of inequivalence between each monomer unit within the trimer. This was confirmed through structural analysis via X-ray crystallography, which provided invaluable insights. It revealed a trimeric species consisting of three monomers featuring the [AuCH(PPh_2_Me)(Ph_2_P)] motif, along with four possible absolute configurations (RRR, RRS, RSR, and SRR). The RRR configuration may be hindered by steric factors, while the remaining three configurations are equivalent due to the inherent structural arrangement and symmetry operations within the metallacycle, allowing interconversion, resulting in only one isomer and its corresponding enantiomer. A comprehensive investigation into the electronic properties of this complex through DFT and TD-DFT studies unveiled its luminescent behavior. This luminescence is attributed to phosphorescence, originating from metal-centered (MC) and ligand-to-metal (LMCT) charge transfer excited states. This work paves the way for new avenues in the development of trimeric structures and holds great promise for innovative applications in materials science and beyond.

## Supporting Information Available

^1^H NMR spectrum of the complex; full (top) and extended (bottom) ESI^+^ mass spectrum of the complex; the molecular structure of the trinuclear [AuCH(PPh_2_Me)(Ph_2_P)]_3_^3+^ cation of the complex; X-ray crystallographic data for complex, and selected bond angles (°) for complex (PDF)
