# Supplementary material for: Isolation of a Cyclic Trinuclear Gold(I) Complex with Metalated Phosphorus Ylides: Synthesis and Structural Properties
Source: Inorg Chem. 2024 Mar 5;63(15):6589–99. doi: 10.1021/acs.inorgchem.3c03740 (PMC11022181; doi:10.1021/acs.inorgchem.3c03740)
Supplement: Supplementary file 1 — ic3c03740_si_001.pdf [file ic3c03740_si_001.pdf]

Supplementary Material (ESI) for Inorganic Chemistry
This journal is (c) The American Chemical Society 2021

Supporting Information for

Isolation of a Cyclic Trinuclear Gold(I) Complex with Metallated Phosphorus Ylides: Synthesis and Structural Properties

Renso Visbal,[a,b,*] Noelia Rosado,[c] Jhon Zapata-Rivera[d] and M. Concepción Gimeno[c,*]

[a]*Facultad de Ciencias Naturales y Exactas, Departamento de Química, Universidad del Valle, A.A. 25360, Cali, Colombia*

[b]*Centro de Excelencia en Nuevos Materiales (CENM), Universidad del Valle, A.A. 25360, Cali, Colombia*

[c]*Departamento de Química Inorgánica, Instituto de Síntesis Química y Catálisis Homogénea (ISQCH) CSIC-Universidad de Zaragoza, 50009 Zaragoza, Spain, email: gimeno@unizar.es*

[d]*Departamento de Química, Facultad de Ciencias Universidad de los Andes A.A 111711, Cra 1 #18A-12, Bogotá, Colombia, email: renso.visbal@correounivalle.edu.co*

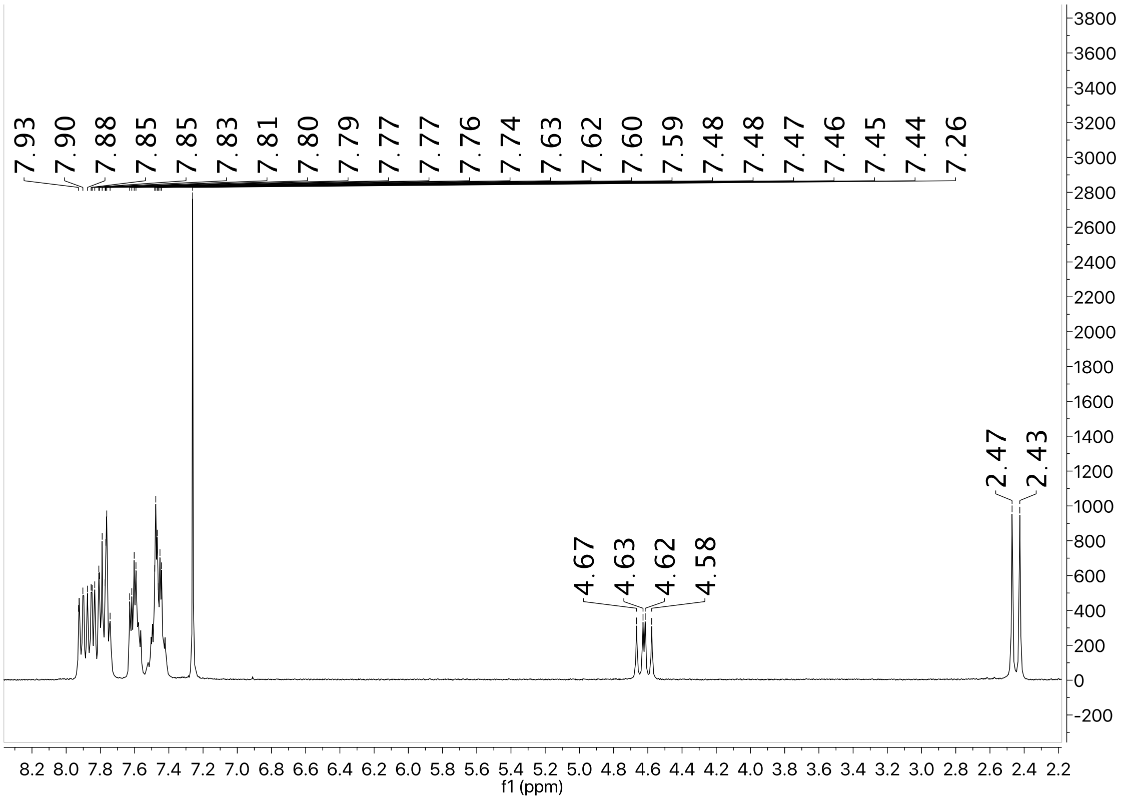

Figure S1. ^{1}H nmr spectrum of complex **1**.

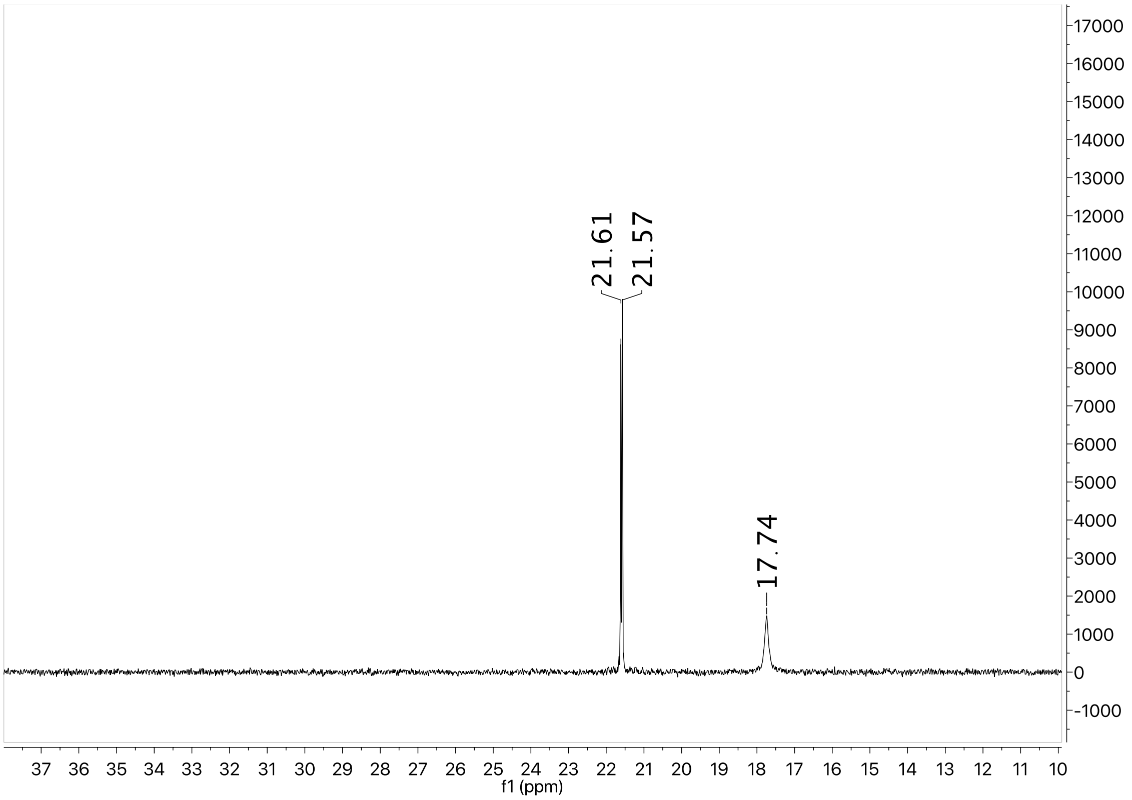

Figure S2. ^{31}P-{^{1}H} nmr spectrum of complex **1**.

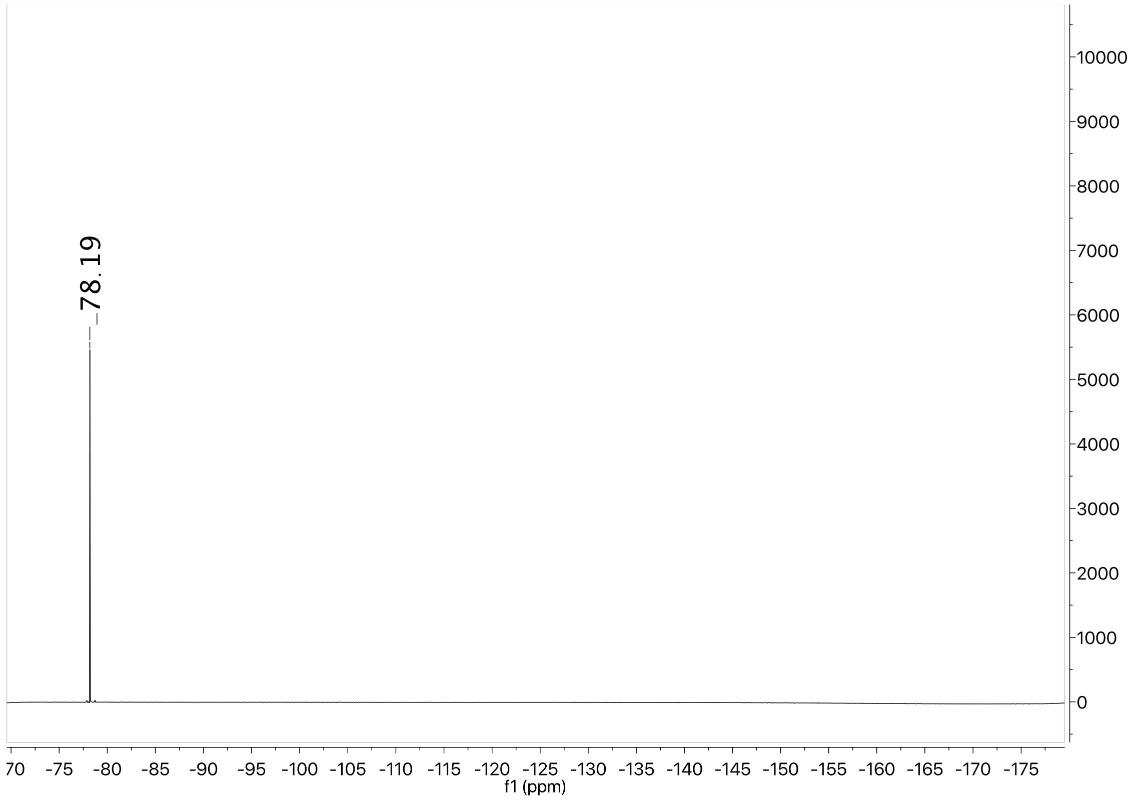

Figure S3. ^{19}F nmr spectrum of complex **1**.

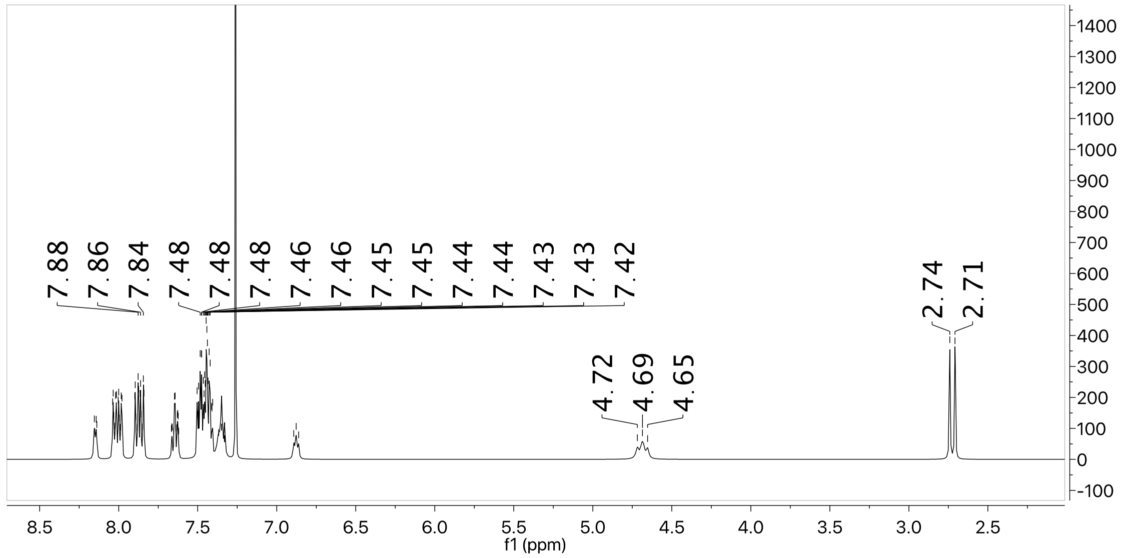

Figure S4. ^{1}H nmr spectrum of complex **2**.

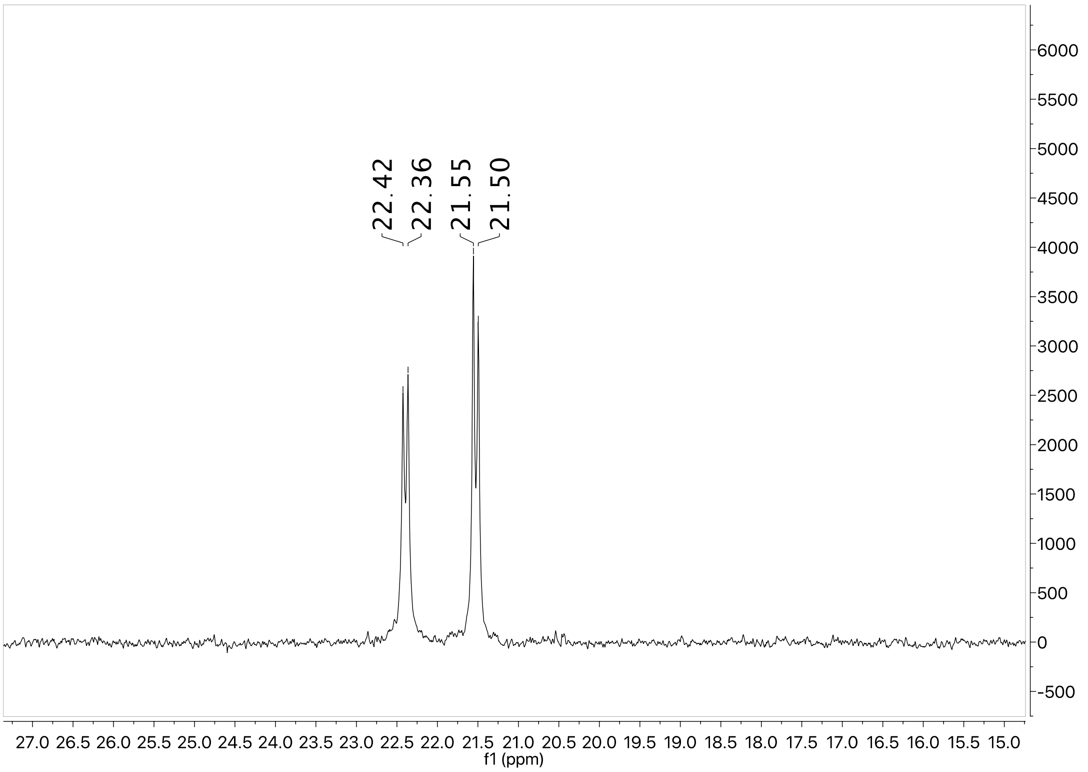

Figure S5. ^{31}P-{^{1}H} nmr spectrum of complex **2**.

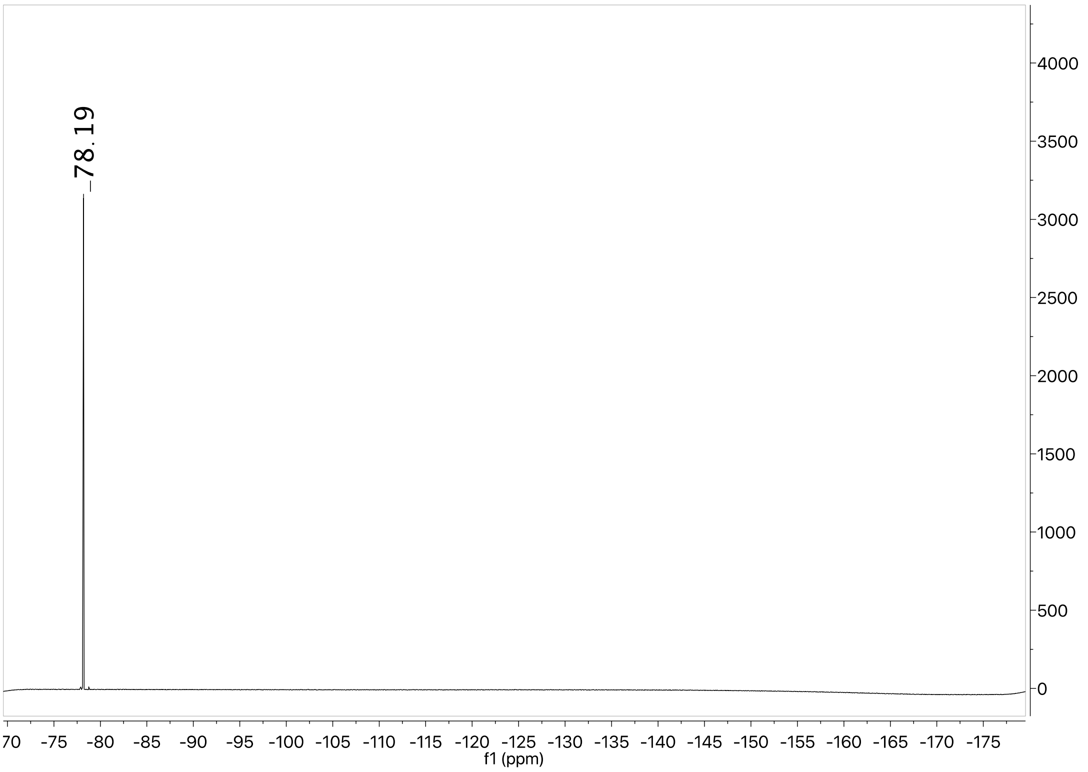

Figure S6. ^{19}F nmr spectrum of complex **2**.

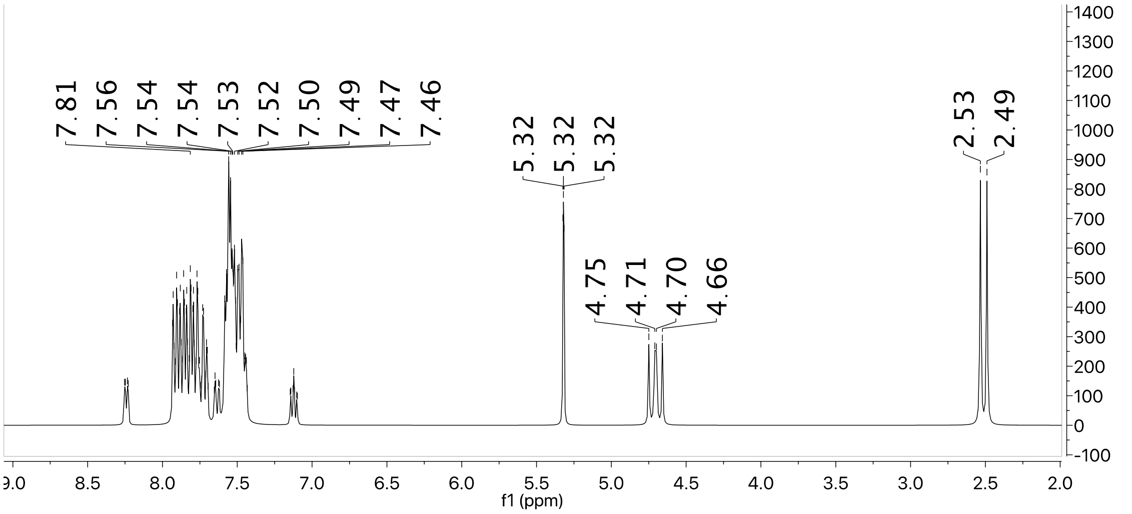

Figure S7. ^{1}H nmr spectrum of complex **3**.

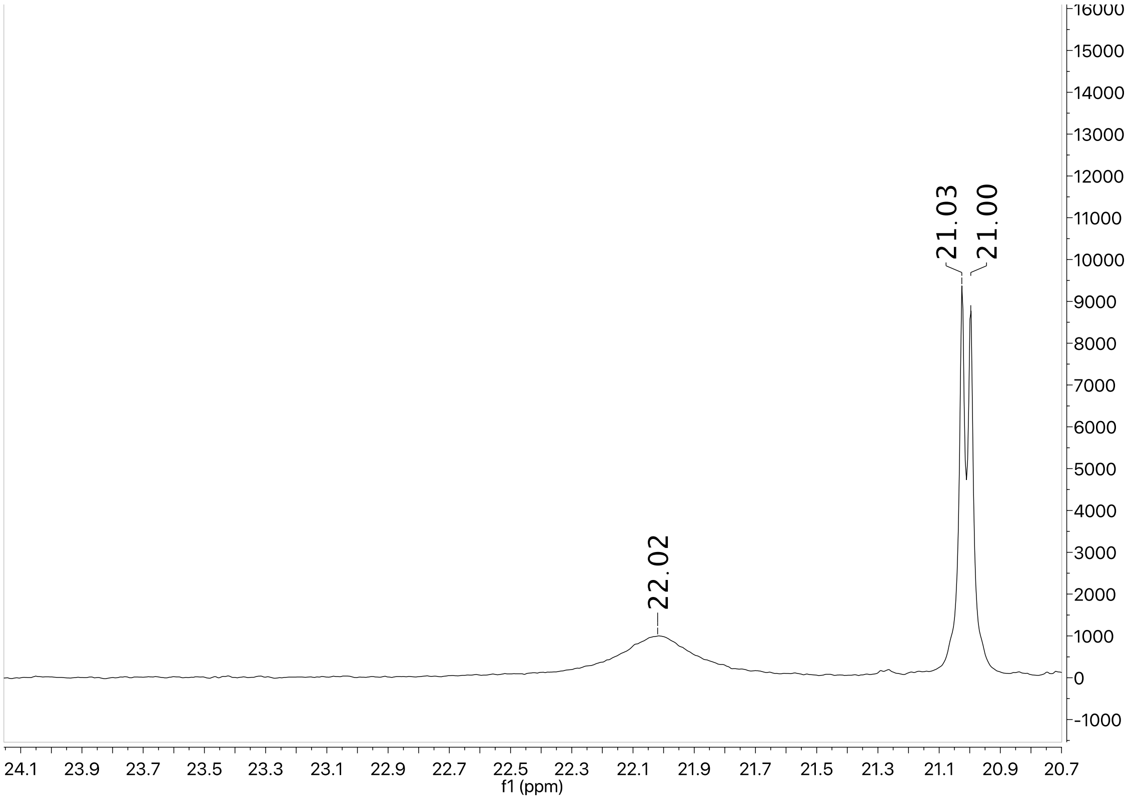

Figure S8. ^{31}P-{^{1}H} nmr spectrum of complex **3**.

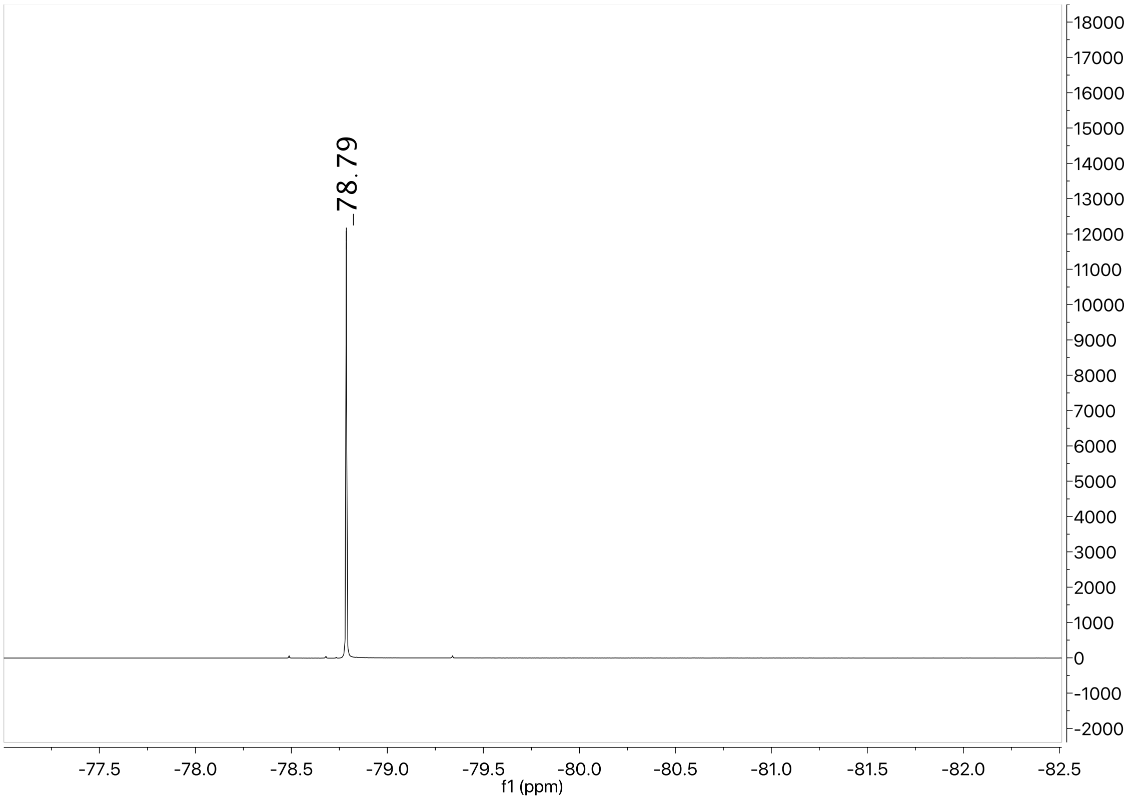

Figure S9. ^{19}F nmr spectrum of complex **3**.

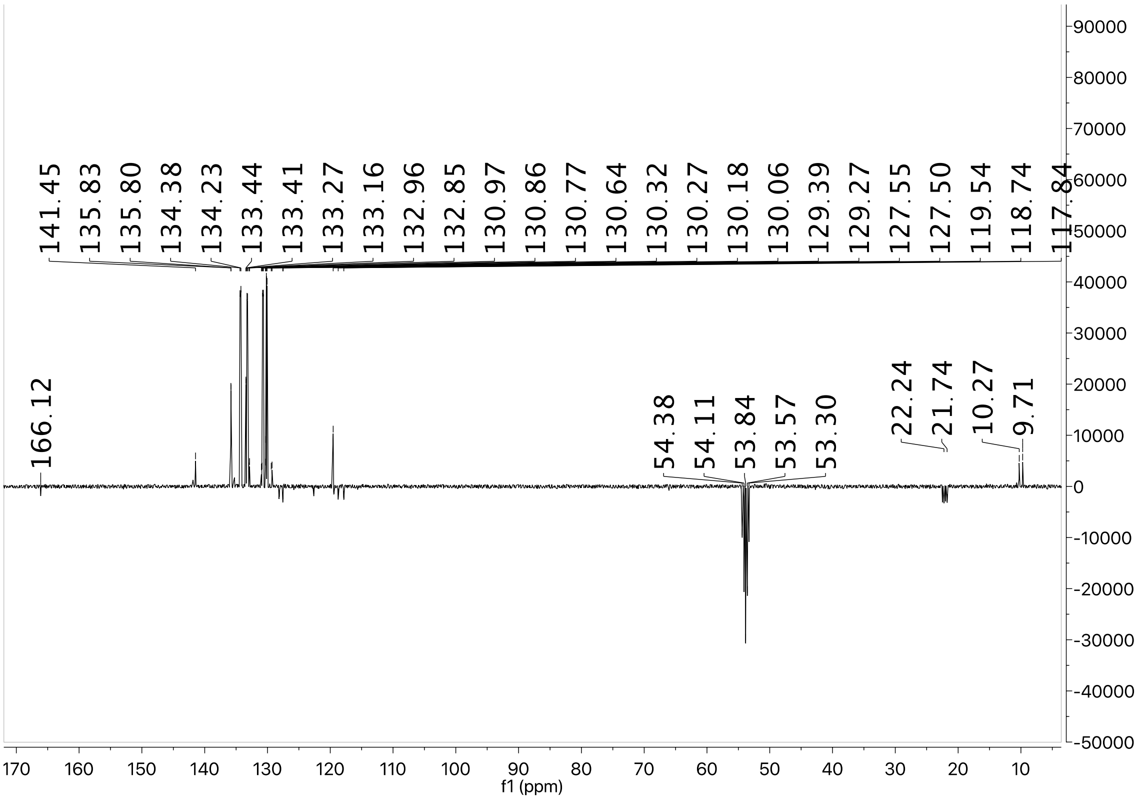

Figure S10. ^{13}C-{^{1}H} nmr spectrum of complex **3**.

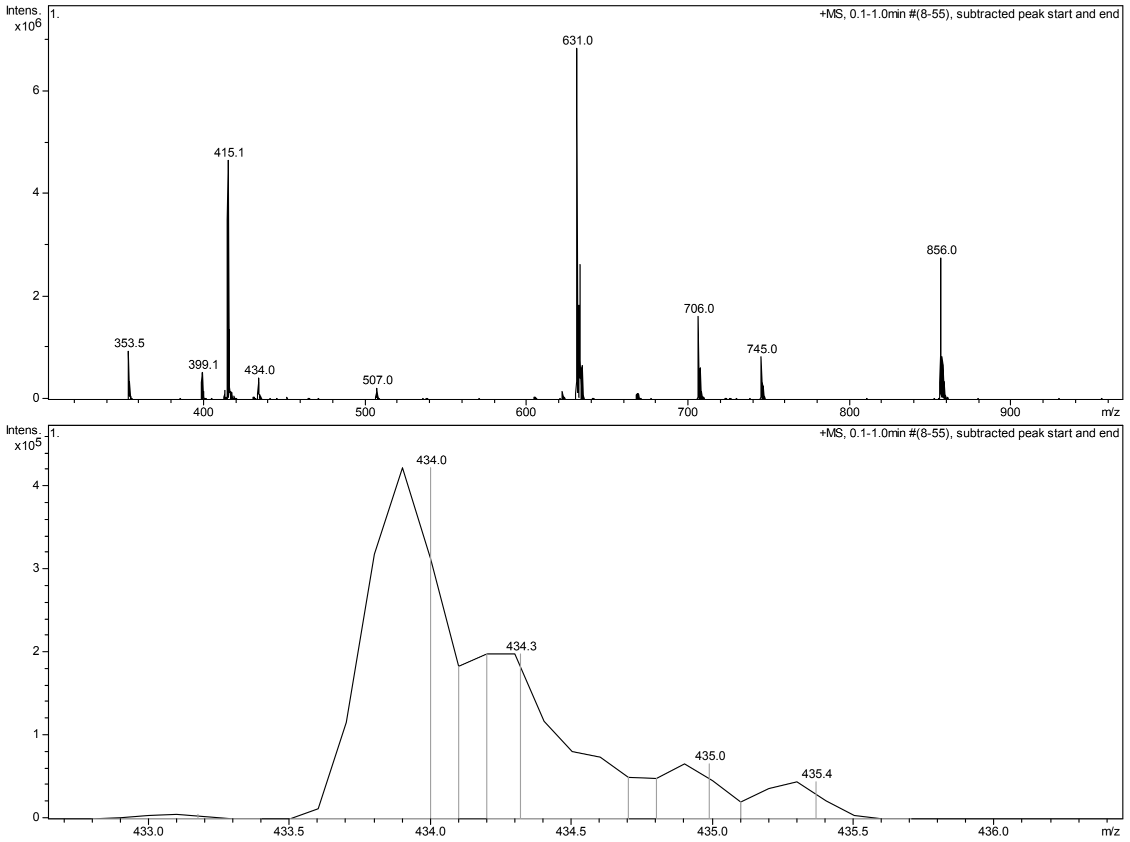

Figure S11. Full (top) and extended (bottom) ESI^+ mass spectrum of complex **3**.

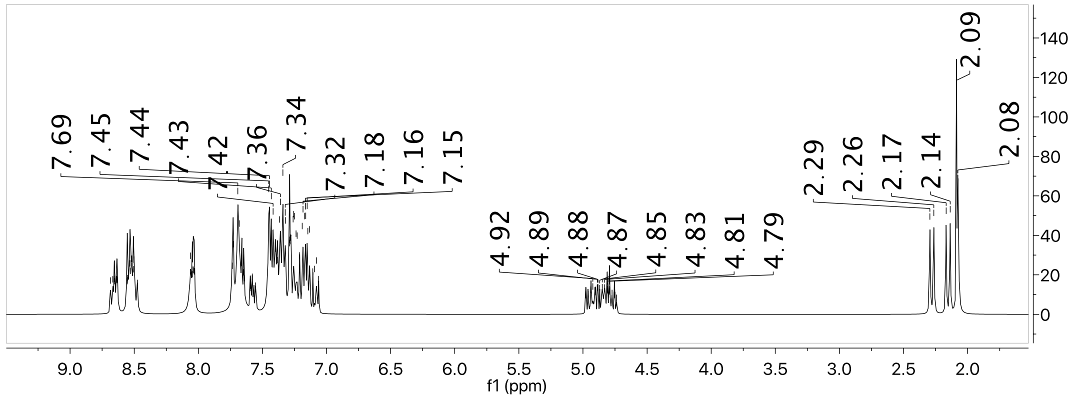

Figure S12. ^1H nmr spectrum of complex **4**.

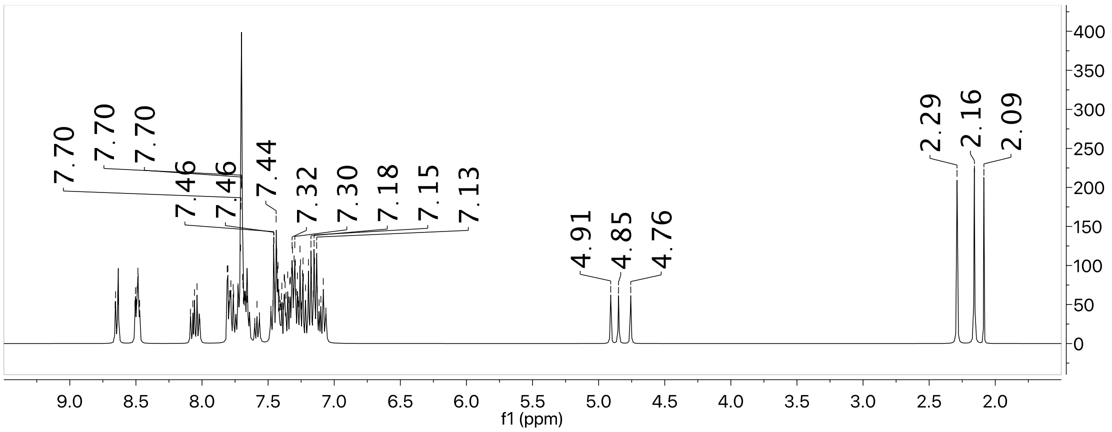

Figure S13. $^{1}H-\{^{31}P\}$ nmr spectrum of complex **4**.

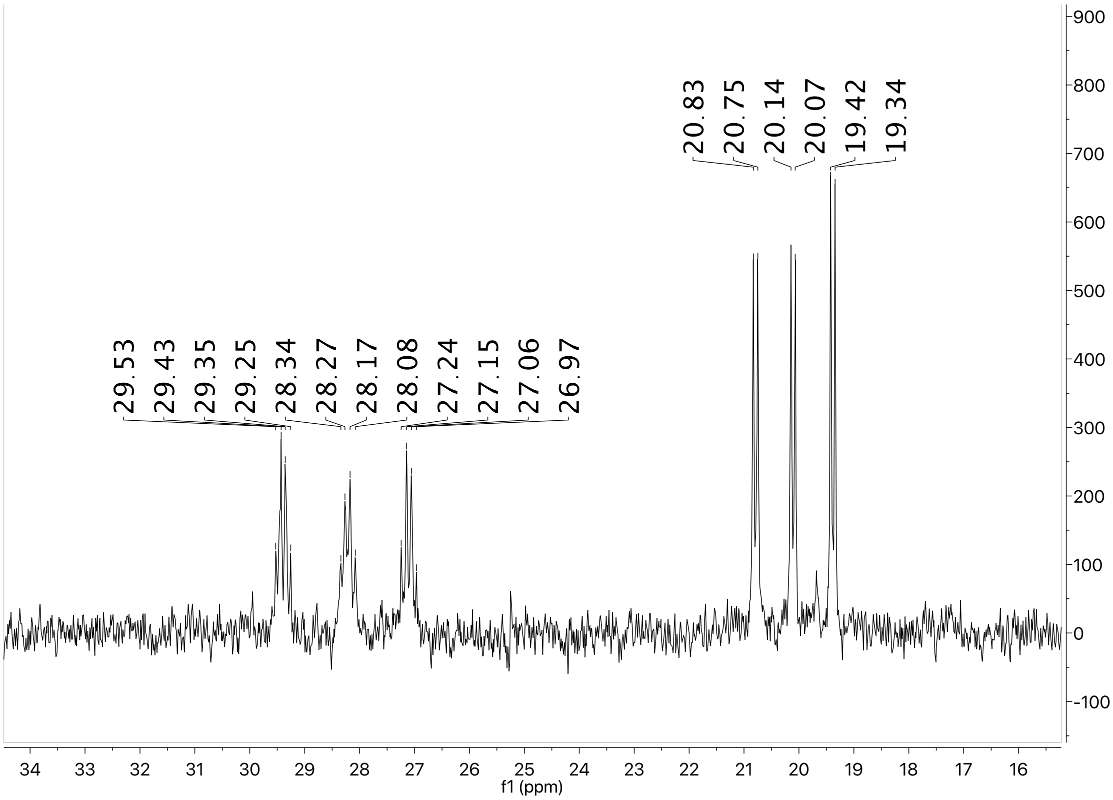

Figure S14. $^{31}P-\{^{1}H\}$ nmr spectrum of complex **4**.

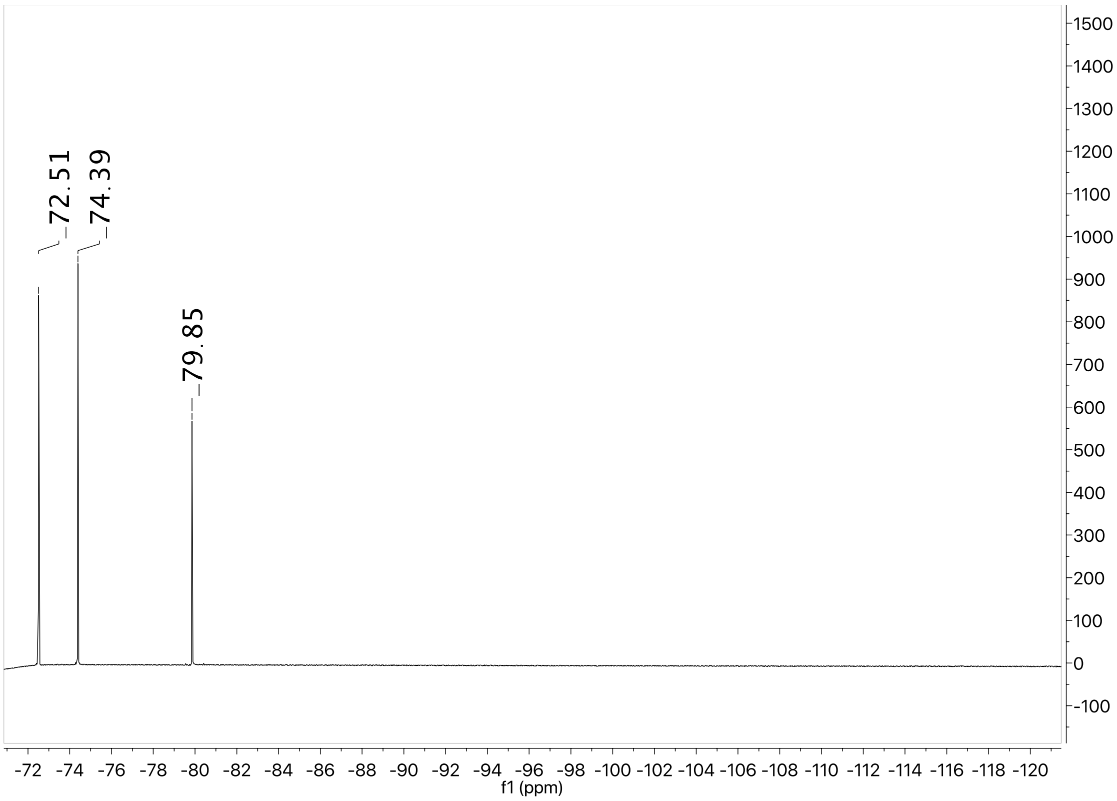

Figure S15. ^{19}F nmr spectrum of complex **4**.

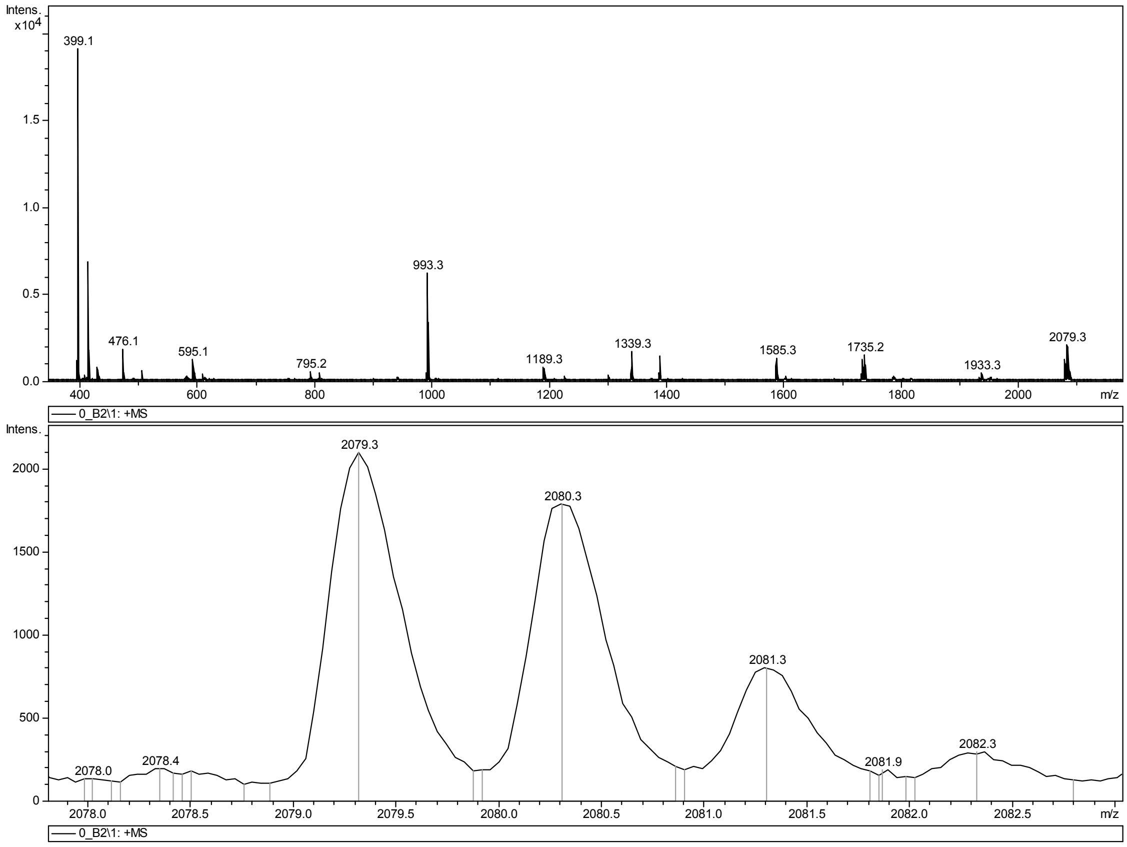

Figure S16. Full (top) and extended (bottom) MALDI-TOF mass spectrum of complex **4** with hexafluorophosphate and trifluoromethylsulfonate as counteranions.

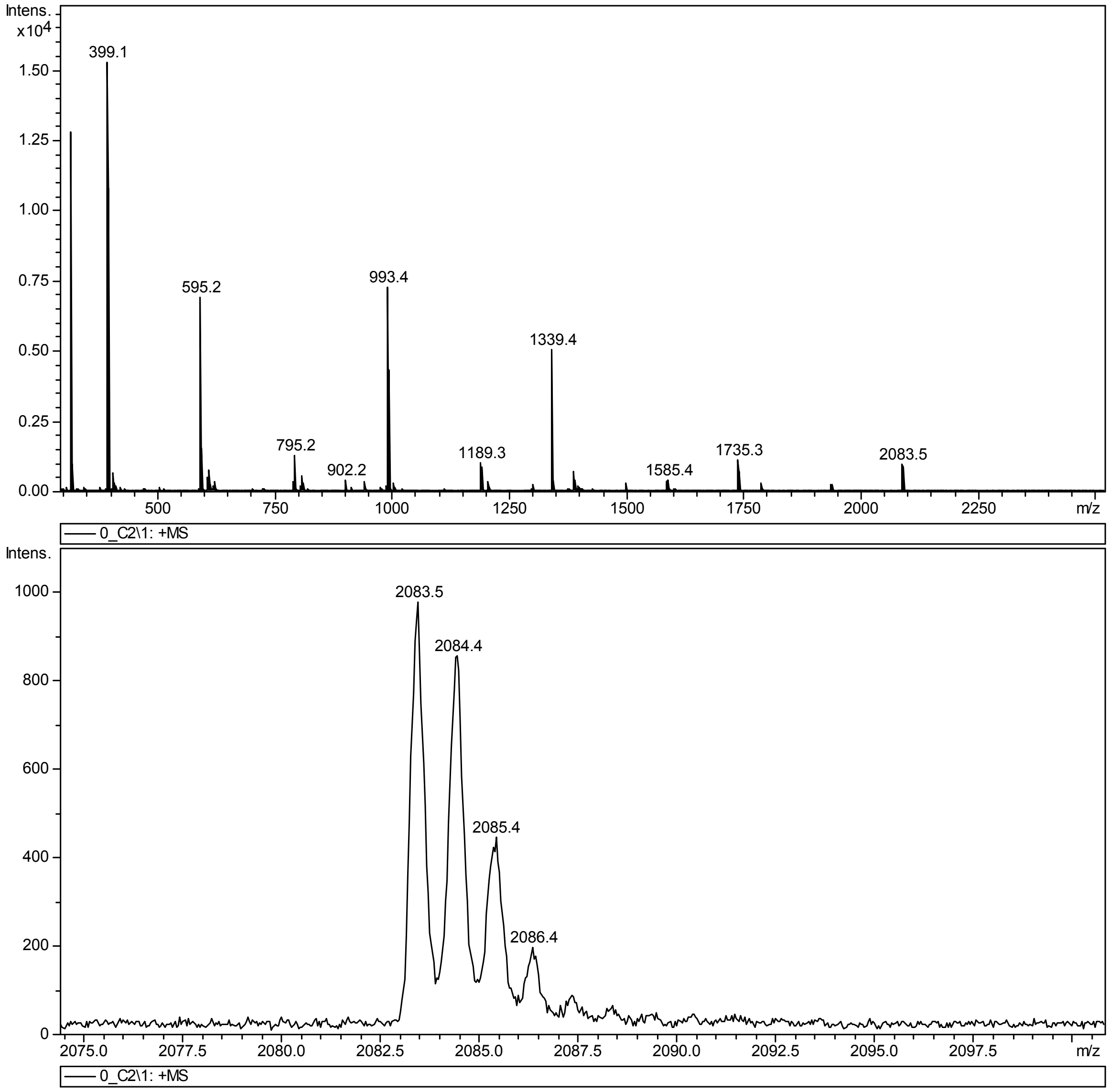

Figure S17. Full (top) and extended (bottom) MALDI-TOF mass spectrum of complex **4** with trifluoromethylsulfonate as counteranions.

Crystallographic data. Crystal Structure Determinations. Crystals were mounted in inert oil on glass fibers and transferred to the cold gas stream of an Xcalibur Oxford Diffraction diffractometer or Bruker Apex Duo equipped with low-temperature attachments. Data were collected using monochromated Mo Kα radiation (λ = 0.71073 Å). The scan type was ω. Absorption corrections based on multiple scans were applied with the program SADABS,[1] or using spherical harmonics implemented in SCALE3 ABSPACK scaling algorithm.[2] The structures were solved with the ShelXS structure solution program using direct methods and by using Olex2 as the graphical interface.[3]

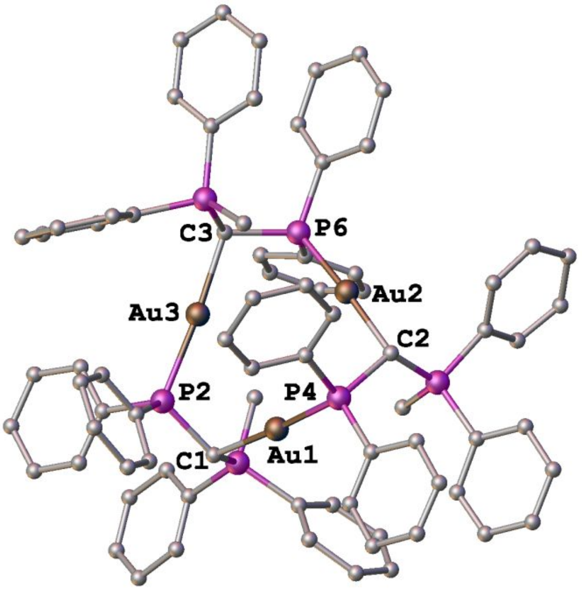

Figure S18. Molecular structure of the trinuclear $[AuCH(PPh_2Me)(Ph_2P)]_3^{3+}$ cation of complex **4**. Hydrogen atoms, anions and solvent molecules are omitted for clarity.

Table S1. X-ray Crystallographic data for complex **4**.

Empirical formula	$C_{82}H_{78}Au_3F_{15}O_4P_8S$
Formula weight	2283.16
Temperature/K	130.15
Crystal system	triclinic
Space group	P-1
a/Å	15.049(3)
b/Å	18.035(4)
c/Å	18.424(4)
α/°	77.48(3)
β/°	87.39(3)
γ/°	74.10(3)
Volume/Å^3	4694.1(18)
Z	2
ρ_{calc}g/cm^3	1.615
μ/mm^{-1}	4.909
F(000)	2220.0
Crystal size/mm^3	0.22 × 0.17 × 0.12
Radiation	MoKα (λ = 0.71073)
2Θ range for data collection/°	8.306 to 57.786
Index ranges	-20 ≤ h ≤ 19, -23 ≤ k ≤ 24, -24 ≤ l ≤ 24
Reflections collected	139848
Independent reflections	22732 [R_{int} = 0.0962, R_{sigma} = 0.0827]
Data/restraints/parameters	22732/57/957
Goodness-of-fit on F^2	1.039
Final R indexes [I>=2σ (I)]	R_1 = 0.0621, wR_2 = 0.1460
Final R indexes [all data]	R_1 = 0.1042, wR_2 = 0.1606
Largest diff. peak/hole / e Å^{-3}	8.54/-2.80

Table S2. Selected bond lengths (Å) for complex **4**.

Atom	Atom	Length/Å	Atom	Atom	Length/Å
Au1	P4	2.277(2)	C44	C45	1.374(17)
Au1	C1	2.107(9)	C45	C46	1.350(16)
Au2	P6	2.277(2)	C46	C47	1.384(13)
Au2	C2	2.106(8)	C48	C49	1.387(12)
Au3	P2	2.284(2)	C48	C53	1.388(12)
Au3	C3	2.101(8)	C49	C50	1.407(12)
P1	C1	1.781(8)	C50	C51	1.366(13)
P1	C4	1.786(10)	C51	C52	1.386(14)
P1	C5	1.800(10)	C52	C53	1.380(12)
P1	C11	1.815(9)	C55	C56	1.412(12)
P2	C1	1.831(9)	C55	C60	1.390(12)
P2	C17	1.816(9)	C56	C57	1.381(12)
P2	C23	1.819(10)	C57	C58	1.389(14)
P3	C2	1.789(8)	C58	C59	1.380(14)
P3	C29	1.788(10)	C59	C60	1.381(12)
P3	C30	1.800(9)	C61	C62	1.390(11)
P3	C36	1.796(11)	C61	C66	1.409(12)
P4	C2	1.819(9)	C62	C63	1.393(13)
P4	C42	1.825(9)	C63	C64	1.379(14)
P4	C48	1.821(8)	C64	C65	1.384(14)
P5	C3	1.801(8)	C65	C66	1.411(13)
P5	C54	1.792(8)	C67	C68	1.431(12)
P5	C55	1.807(8)	C67	C72	1.378(12)
P5	C61	1.791(8)	C68	C69	1.404(14)
P6	C3	1.827(8)	C69	C70	1.341(16)
P6	C67	1.818(9)	C70	C71	1.412(16)
P6	C73	1.810(8)	C71	C72	1.381(14)
C5	C6	1.340(16)	C73	C74	1.374(13)
C5	C10	1.355(17)	C73	C78	1.399(13)
C6	C7	1.42(2)	C74	C75	1.384(14)
C7	C8	1.36(2)	C75	C76	1.355(18)
C8	C9	1.37(2)	C76	C77	1.376(18)
C9	C10	1.42(2)	C77	C78	1.405(14)
C11	C12	1.388(15)	C79	S1	1.464(15)
C11	C16	1.373(15)	C79	F1	1.488(17)
C12	C13	1.399(15)	C79	F2	1.408(16)
C13	C14	1.40(2)	C79	F3	1.480(15)
C14	C15	1.37(2)	S1	O1	1.405(7)
C15	C16	1.380(15)	S1	O2	1.392(8)
C17	C18	1.385(13)	S1	O3	1.437(7)
C17	C22	1.393(13)	P7	F4	1.507(11)
C18	C19	1.395(15)	P7	F5	1.630(12)
C19	C20	1.385(18)	P7	F6	1.484(11)
C20	C21	1.403(19)	P7	F7	1.643(13)
C21	C22	1.359(15)	P7	F8	1.557(9)
C23	C24	1.387(13)	P7	F9	1.677(14)
C23	C28	1.417(14)	P9	F13	1.41(3)
C24	C25	1.398(15)	P9	F14	1.53(3)
C25	C26	1.378(16)	P9	F15	1.59(4)

C26	C27	1.364(15)	P9	F16	1.55(2)
C27	C28	1.371(14)	P9	F17	1.49(3)
C30	C31	1.392(14)	P9	F18	1.41(2)
C30	C35	1.400(13)	O4	C80	1.16(2)
C31	C32	1.377(15)	C80	C81	1.48(3)
C32	C33	1.370(19)	C80	C82	1.47(4)
C33	C34	1.37(2)	O5	C83	1.33(3)
C34	C35	1.402(15)	C83	C84	1.31(3)
C36	C37	1.397(16)	C83	C85	1.55(4)
C36	C41	1.378(14)	P8	F10	1.632(3)
C37	C38	1.38(2)	P8	F10[1]	1.632(3)
C38	C39	1.40(2)	P8	F12	1.600(5)
C39	C40	1.42(2)	P8	F12[1]	1.600(5)
C40	C41	1.349(18)	P8	F11[1]	1.596(5)
C42	C43	1.372(13)	P8	F11	1.596(5)
C42	C47	1.381(13)			

[1]1-X,1-Y,1-Z

Table S3. Selected bond angles (°) for complex **4**.

Atom	Atom	Atom	Angle/°	Atom	Atom	Atom	Angle/°
C1	Au1	P2	36.3(2)	C45	C46	C47	120.9(10)
C1	Au1	P4	175.2(2)	C42	C47	C46	119.7(10)
C2	Au2	P6	173.1(2)	C49	C48	P4	121.9(6)
C3	Au3	P2	176.0(2)	C49	C48	C53	119.1(7)
C1	P1	C4	110.8(4)	C53	C48	P4	118.8(7)
C1	P1	C5	113.4(4)	C48	C49	C50	119.0(8)
C1	P1	C11	108.2(5)	C51	C50	C49	121.1(9)
C4	P1	C5	108.4(5)	C50	C51	C52	119.9(8)
C4	P1	C11	108.7(5)	C53	C52	C51	119.3(9)
C5	P1	C11	107.1(4)	C52	C53	C48	121.6(9)
Au3	P2	Au1	83.05(7)	C56	C55	P5	120.4(6)
C1	P2	Au1	43.0(3)	C60	C55	P5	120.0(6)
C1	P2	Au3	113.4(3)	C60	C55	C56	119.5(7)
C17	P2	Au1	154.1(3)	C57	C56	C55	119.6(9)
C17	P2	Au3	113.3(3)	C56	C57	C58	120.3(9)
C17	P2	C1	111.2(4)	C59	C58	C57	119.9(8)
C17	P2	C23	103.3(4)	C58	C59	C60	120.8(9)
C23	P2	Au1	88.6(3)	C59	C60	C55	119.8(9)
C23	P2	Au3	110.0(3)	C62	C61	P5	119.7(6)
C23	P2	C1	104.9(4)	C62	C61	C66	119.6(8)
C2	P3	C30	113.1(4)	C66	C61	P5	120.6(6)
C2	P3	C36	108.1(4)	C61	C62	C63	120.7(9)
C29	P3	C2	111.6(4)	C64	C63	C62	120.1(9)
C29	P3	C30	107.3(4)	C63	C64	C65	120.2(9)
C29	P3	C36	108.9(5)	C64	C65	C66	120.6(9)
C36	P3	C30	107.6(5)	C61	C66	C65	118.8(8)
C2	P4	Au1	113.1(3)	C68	C67	P6	118.5(7)
C2	P4	C42	110.6(4)	C72	C67	P6	121.6(7)
C2	P4	C48	104.3(4)	C72	C67	C68	119.9(8)
C42	P4	Au1	114.2(3)	C69	C68	C67	117.6(9)

C48	P4	Au1	110.3(3)	C70	C69	C68	121.7(10)
C48	P4	C42	103.5(4)	C69	C70	C71	120.6(10)
C3	P5	C55	107.0(4)	C72	C71	C70	119.3(10)
C54	P5	C3	112.3(4)	C67	C72	C71	120.7(10)
C54	P5	C55	108.6(4)	C74	C73	P6	119.1(7)
C61	P5	C3	111.7(4)	C74	C73	C78	118.4(8)
C61	P5	C54	109.4(4)	C78	C73	P6	122.4(7)
C61	P5	C55	107.6(4)	C73	C74	C75	121.9(11)
C3	P6	Au2	113.8(3)	C76	C75	C74	120.3(11)
C67	P6	Au2	117.7(3)	C75	C76	C77	119.3(10)
C67	P6	C3	109.1(4)	C76	C77	C78	121.5(11)
C73	P6	Au2	107.4(3)	C73	C78	C77	118.6(10)
C73	P6	C3	104.8(4)	S1	C79	F1	119.5(11)
C73	P6	C67	102.5(4)	S1	C79	F3	122.2(10)
P1	C1	Au1	112.1(4)	F2	C79	S1	124.4(11)
P1	C1	P2	116.4(5)	F2	C79	F1	94.5(10)
P2	C1	Au1	100.6(4)	F2	C79	F3	95.3(10)
P3	C2	Au2	106.7(4)	F3	C79	F1	93.3(10)
P3	C2	P4	117.9(4)	O1	S1	C79	99.0(7)
P4	C2	Au2	105.5(4)	O1	S1	O3	115.5(5)
P5	C3	Au3	108.0(4)	O2	S1	C79	105.5(7)
P5	C3	P6	114.6(5)	O2	S1	O1	115.8(5)
P6	C3	Au3	108.2(4)	O2	S1	O3	117.6(6)
C6	C5	P1	121.6(8)	O3	S1	C79	99.0(7)
C6	C5	C10	120.4(11)	F4	P7	F5	92.7(6)
C10	C5	P1	118.0(10)	F4	P7	F7	172.6(6)
C5	C6	C7	121.0(13)	F4	P7	F8	90.4(5)
C8	C7	C6	118.0(17)	F4	P7	F9	94.2(6)
C7	C8	C9	122.1(17)	F5	P7	F7	82.4(6)
C8	C9	C10	118.1(16)	F5	P7	F9	83.3(6)
C5	C10	C9	120.4(15)	F6	P7	F4	98.8(6)
C12	C11	P1	120.5(8)	F6	P7	F5	94.0(6)
C16	C11	P1	118.4(8)	F6	P7	F7	87.2(6)
C16	C11	C12	121.0(9)	F6	P7	F8	94.3(5)
C11	C12	C13	119.3(11)	F6	P7	F9	166.8(7)
C14	C13	C12	118.0(13)	F7	P7	F9	79.7(7)
C15	C14	C13	122.4(12)	F8	P7	F5	170.6(6)
C14	C15	C16	118.9(12)	F8	P7	F7	93.6(6)
C11	C16	C15	120.3(12)	F8	P7	F9	87.7(6)
C18	C17	P2	120.1(7)	F13	P9	F14	84.4(18)
C18	C17	C22	118.9(9)	F13	P9	F15	175(2)
C22	C17	P2	120.9(8)	F13	P9	F16	93.0(17)
C17	C18	C19	120.4(10)	F13	P9	F17	90.3(18)
C20	C19	C18	119.6(12)	F14	P9	F15	91.0(19)
C19	C20	C21	120.1(11)	F14	P9	F16	177.1(16)
C22	C21	C20	119.4(12)	F16	P9	F15	91.6(17)
C21	C22	C17	121.7(11)	F17	P9	F14	100.9(18)
C24	C23	P2	116.6(8)	F17	P9	F15	89.3(18)
C24	C23	C28	118.7(9)	F17	P9	F16	80.2(14)
C28	C23	P2	124.6(7)	F18	P9	F13	94.4(17)
C23	C24	C25	119.5(11)	F18	P9	F14	81.2(16)

C26	C25	C24	120.7(10)	F18	P9	F15	86.2(17)
C27	C26	C25	119.9(10)	F18	P9	F16	97.9(14)
C26	C27	C28	120.9(11)	F18	P9	F17	175.1(16)
C27	C28	C23	120.2(10)	O4	C80	C81	127(2)
C31	C30	P3	121.0(8)	O4	C80	C82	117(2)
C31	C30	C35	120.6(9)	C82	C80	C81	116(2)
C35	C30	P3	118.2(8)	O5	C83	C85	121(2)
C32	C31	C30	119.7(12)	C84	C83	O5	119(3)
C33	C32	C31	119.9(13)	C84	C83	C85	120(2)
C32	C33	C34	121.6(11)	F10[1]	P8	F10	180.0(8)
C33	C34	C35	119.7(12)	F12[1]	P8	F10[1]	73.3(9)
C30	C35	C34	118.5(11)	F12[1]	P8	F10	106.7(9)
C37	C36	P3	117.1(9)	F12	P8	F10	73.3(9)
C41	C36	P3	122.4(7)	F12	P8	F10[1]	106.7(9)
C41	C36	C37	120.3(11)	F12	P8	F12[1]	180.0
C38	C37	C36	118.4(15)	F11	P8	F10	74.5(11)
C37	C38	C39	122.2(19)	F11[1]	P8	F10[1]	74.5(11)
C38	C39	C40	116.6(19)	F11	P8	F10[1]	105.5(11)
C41	C40	C39	121.4(16)	F11[1]	P8	F10	105.5(11)
C40	C41	C36	120.7(11)	F11	P8	F12[1]	84.7(13)
C43	C42	P4	119.8(7)	F11[1]	P8	F12	84.6(13)
C43	C42	C47	119.0(9)	F11[1]	P8	F12[1]	95.3(13)
C47	C42	P4	121.2(7)	F11	P8	F12	95.3(13)
C42	C43	C44	121.1(11)	F11[1]	P8	F11	180.0
C45	C44	C43	118.7(11)				

[1]1-X,1-Y,1-Z

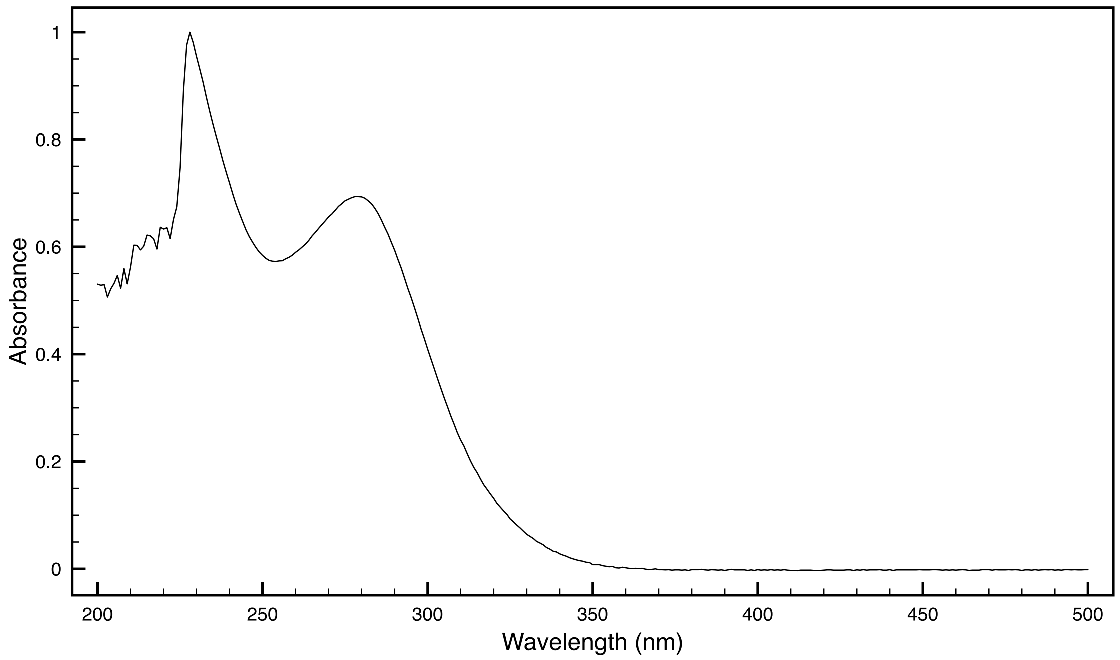

Figure S19. Electronic spectrum of complex **2** in acetone (10^{-3} M).

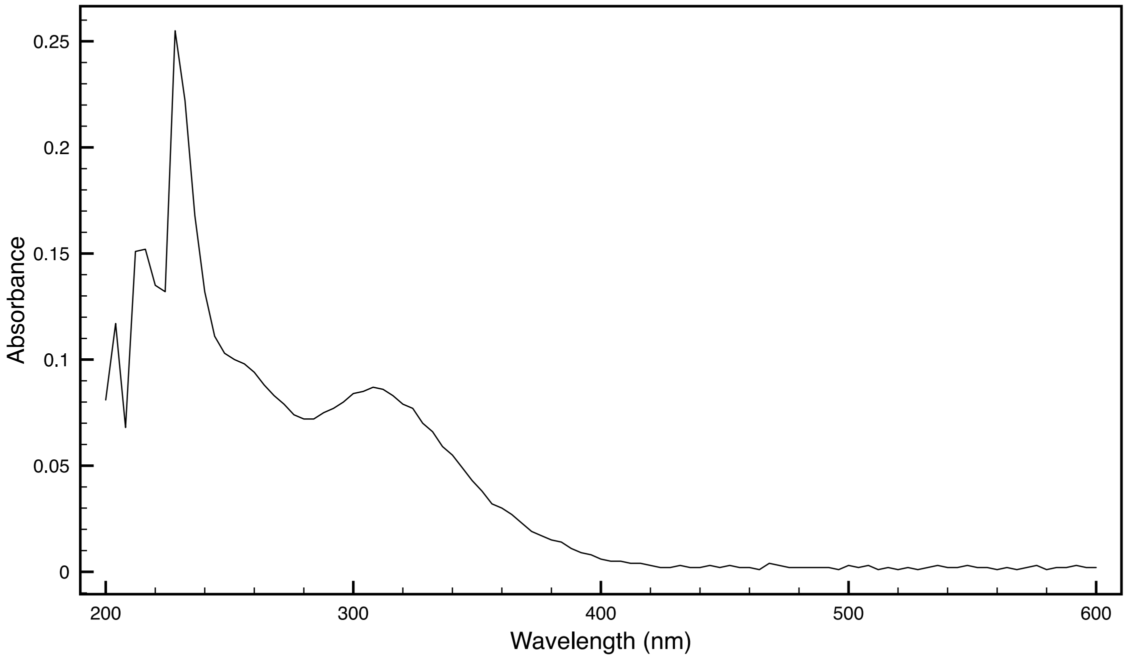

Figure S20. Electronic spectrum of complex **3** in acetone (10^{-3} M).

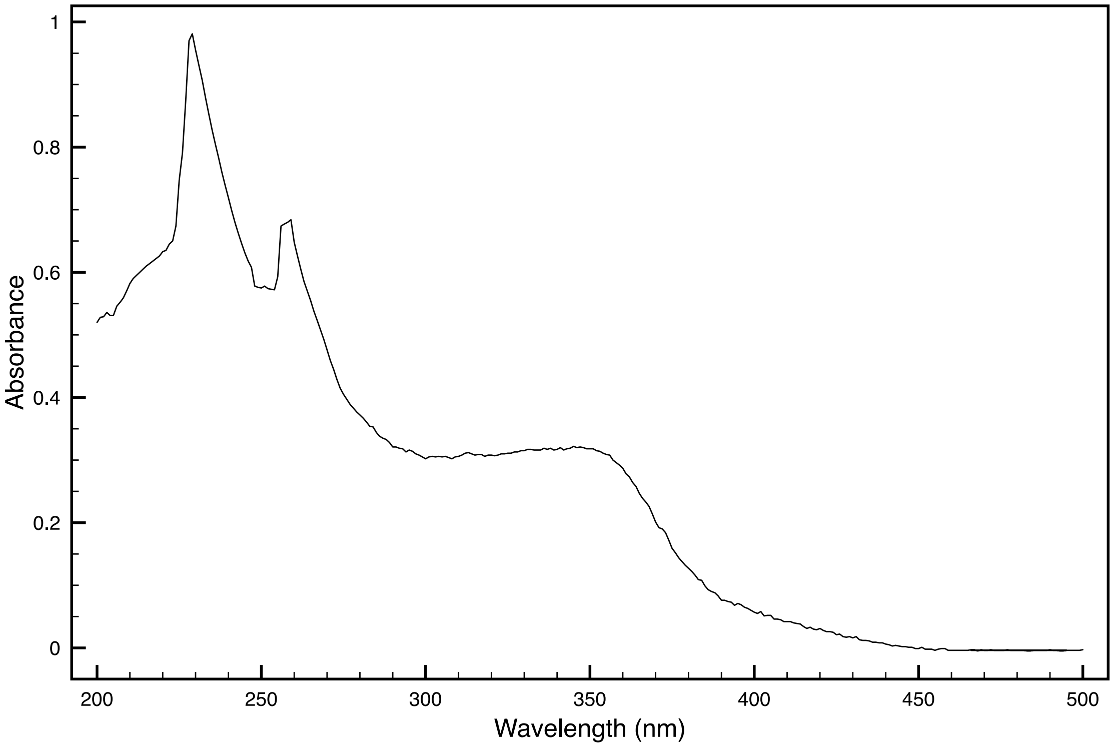

Figure S21. Electronic spectrum of complex **4** in acetone (10^{-3} M).

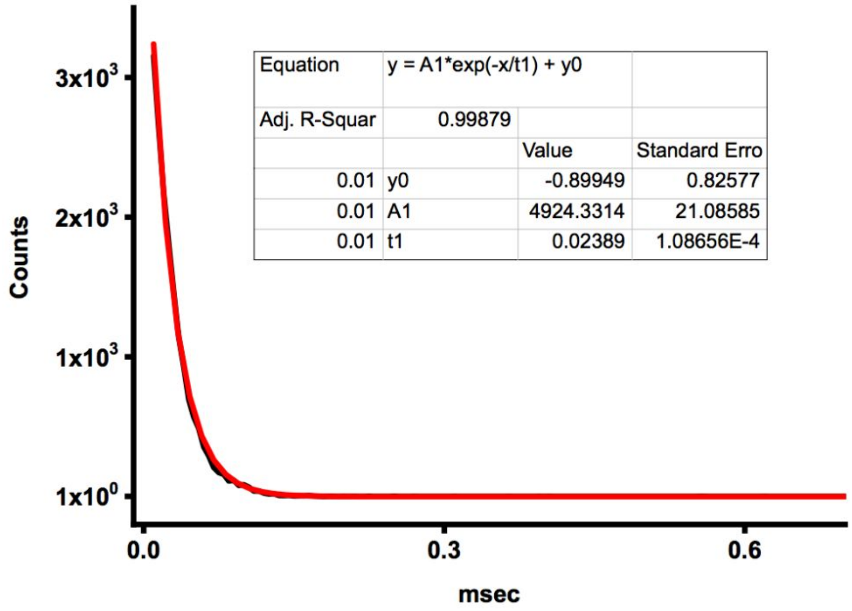

Figure S22. Emission lifetime measured for complex **4** in acetone (0.5 mg/25 ml).

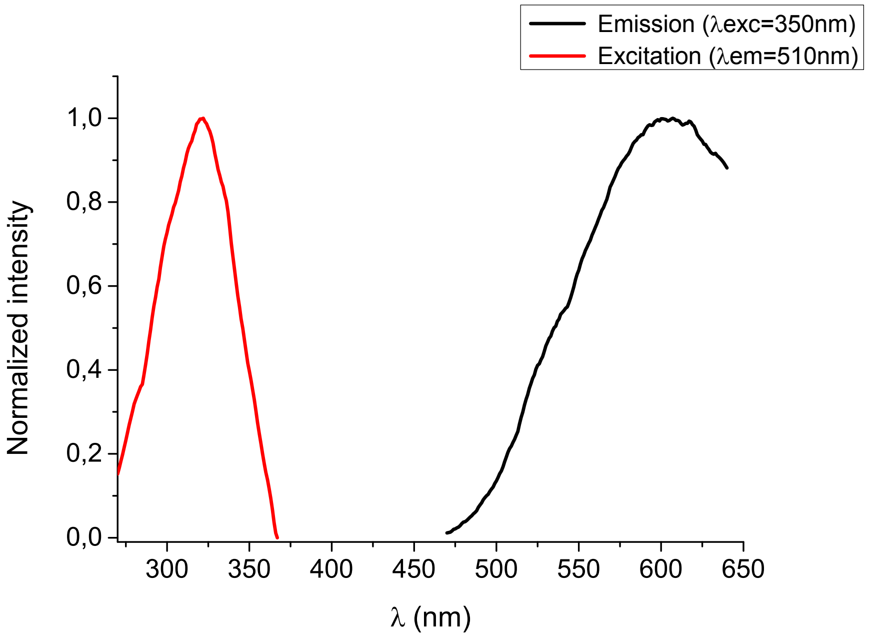

Figure S23. Excitation (left) and emission (right) spectra of complex **4** in the solid state at room temperature.

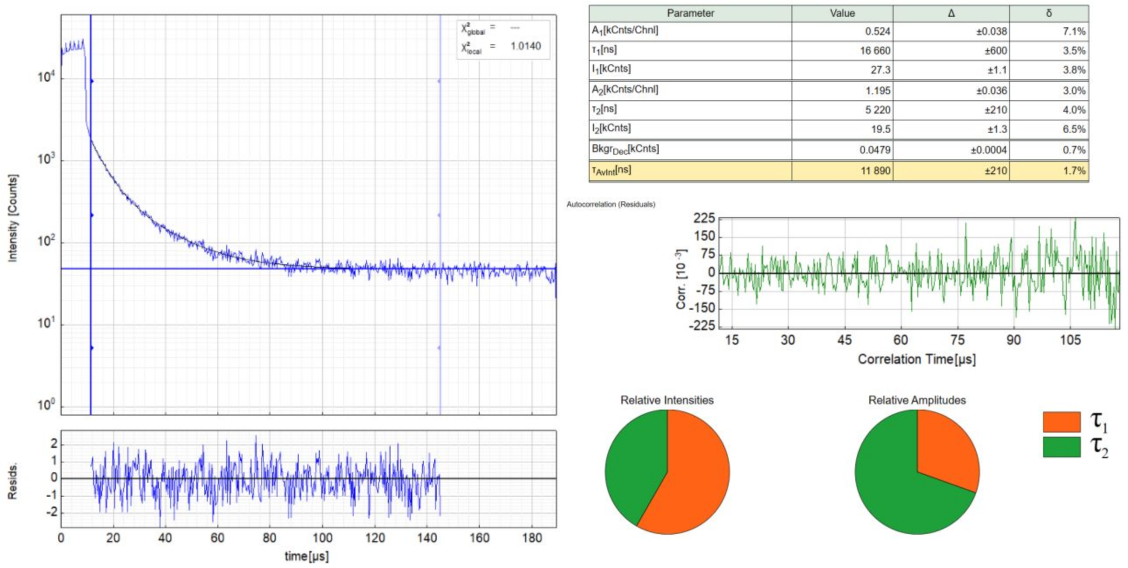

Figure S24. Emission lifetime of complex **4** in the solid state at room temperature.

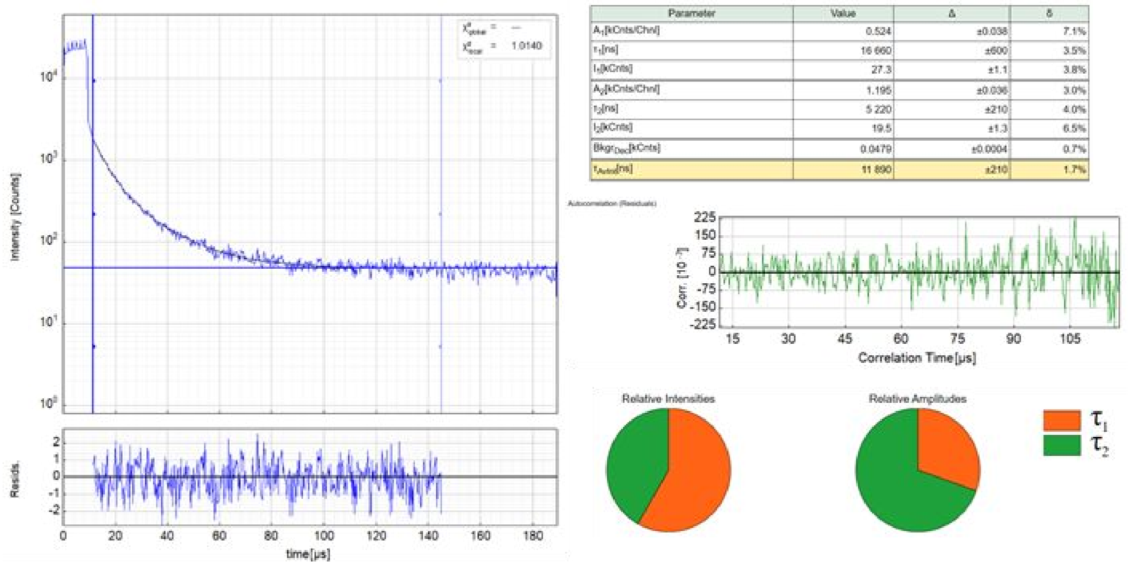

Figure S25. Emission lifetime of complex **4** in the solid state at 77 K.

Computational details

In order to evaluate the electronic structure and the low-lying energies of the CTC **4**, some calculations have been carried out on the framework of Density Functional Theory. Using the crystallographic structure as starting point, single point and geometry optimization calculations have been performed with PBE0 functional.[4] This functional has been used in the evaluation phochemical properties of other Au(I) complexes producing reliable results.[5] In all calculations, an Effective Core Potential (ECP)[6] version of the def2-TZVP basis set has been used for Au and P atoms, whereas the def2-SVP quality for remaining atoms,[7,8] as implemented in the ORCA 5.0.3 package.[9,10] The resolution of identity approach has also been considered by using the def2/J auxiliary basis set for Coulomb integrals computation.[11] The solvent effect was also added by the Conductor-like Polarizable Continuum Model (CPCM).[12] In the simulations we have used acetone as solvent (dielectric constant = 20.7 and refractive index = 1.359). The threshold for the energy convergence in the self-consistent field procedure was $1x10^{-8}$ a.u. No negative normal modes were obtained by analytical frequency calculations on the optimized geometries. Time Dependent DFT calculations have been also carried out to simulate the absorption spectrum of complex **4**. 200 excited singlet states have been calculated.

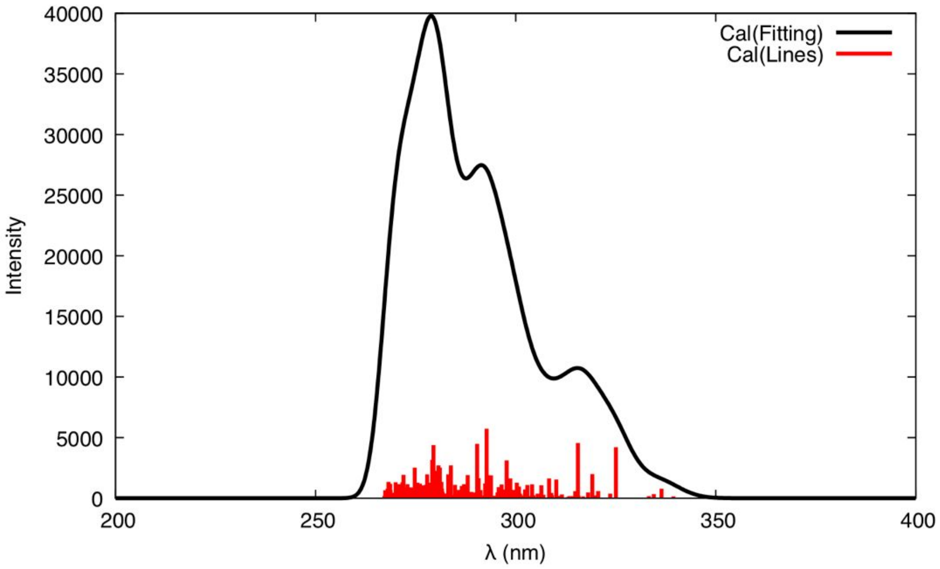

Figure S26. Calculated absorption spectrum via transition electric dipole moments of complex **4**.

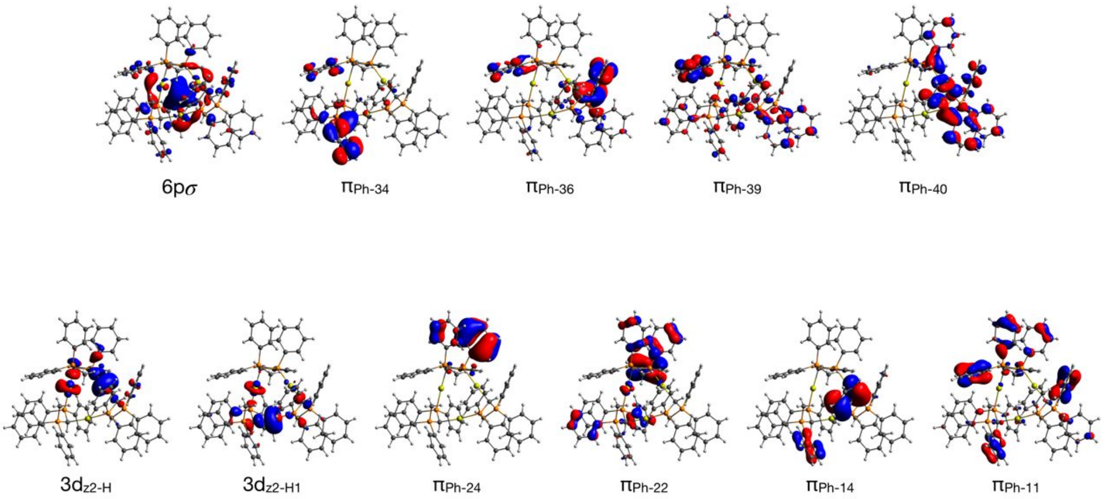

Figure S27. Molecular orbitals involved in the dominant electronic transitions that lead to the studied excited singlet states of complex **4**.

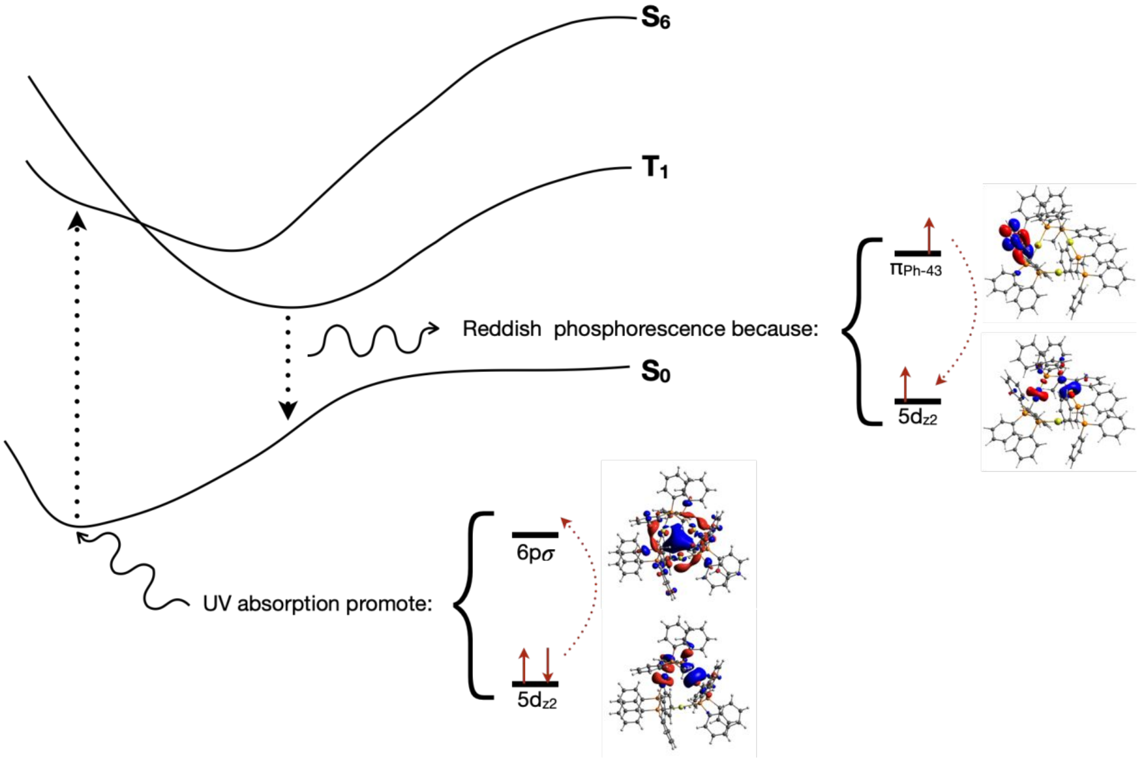

Figure S28. Schematic representation of the absorption and emission of CTC **4**. Molecular orbitals involved in the main transitions are also shown.

References

(1) Sheldrick, G. M. SADABS, Program for Area Detector Adsorption Correction. Institute for Inorganic Chemistry, University of Gottingen: Gottingen, Germany 1996.

(2) Agilent Technologies. CrysAlisPro, Version 1.171.35.11 Multiscan Absorption Correction with SCALE3 ABSPACK Scaling Algorithm.

(3) Dolomanov, O. V.; Bourhis, L. J.; Gildea, R. J.; Howard, J. A. K.; Puschmann, H. OLEX2: A Complete Structure Solution, Refinement and Analysis Program. *J. Appl. Crystallogr.* **2009**, *42* (2), 339–341.

(4) Adamo, C.; Barone, V. Toward Reliable Density Functional Methods without Adjustable Parameters: The PBE0 Model. *J. Chem. Phys.* **1999**, *110* (13), 6158–6170.

(5) Aguiló, E.; Moro, A. J.; Outis, M.; Pina, J.; Sarmento, D.; Seixas De Melo, J. S.; Rodríguez, L.; Lima, J. C. Deactivation Routes in Gold(I) Polypyridyl Complexes: Internal Conversion Vs Fast Intersystem Crossing. *Inorg. Chem.* **2018**, *57* (21), 13423–13430.

(6) Schwerdtfeger, P.; Dolg, M.; Schwarz, W. H. E.; Bowmaker, G. A.; Boyd, P. D. W. Relativistic Effects in Gold Chemistry. I. Diatomic Gold Compounds. *J. Chem. Phys.* **1989**, *91* (3), 1762–1774.

(7) Andrae, D.; Häußermann, U.; Dolg, M.; Stoll, H.; Preuß, H. Energy-Adjusted Ab Initio Pseudopotentials for the Second and Third Row Transition Elements. *Theor. Chim. Acta* **1990**, *77* (2), 123–141.

(8) Weigend, F.; Ahlrichs, R. Balanced Basis Sets of Split Valence, Triple Zeta Valence and Quadruple Zeta Valence Quality for H to Rn: Design and Assessment of Accuracy. *Phys. Chem. Chem. Phys.* **2005**, *7* (18), 3297–3305.

(9) Neese, F. The ORCA Program System. *Wiley Interdiscip. Rev. Comput. Mol. Sci.* **2012**, *2* (1), 73–78.

(10) Neese, F. Software Update: The ORCA Program System, Version 4.0. *Wiley Interdiscip. Rev. Comput. Mol. Sci.* **2018**, *8* (1), 1–6.

(11) Weigend, F. Accurate Coulomb-Fitting Basis Sets for H to Rn. *Phys. Chem. Chem. Phys.* **2006**, *8* (9), 1057–1065.

(12) Barone, V.; Cossi, M. Conductor Solvent Model. *J. Phys. Chem. A* **1998**, *102* (97), 1995–2001.
